# Frailty in individuals with depression, bipolar disorder and anxiety disorders: longitudinal analyses of all-cause mortality

**DOI:** 10.1186/s12916-022-02474-2

**Published:** 2022-08-30

**Authors:** Julian Mutz, Umamah Choudhury, Jinlong Zhao, Alexandru Dregan

**Affiliations:** 1 Social, Genetic and Developmental Psychiatry Centre, Institute of Psychiatry, Psychology & Neuroscience, King’s College London, Memory Lane, London, SE5 8AF UK; 2 Department of Basic & Clinical Neuroscience, Institute of Psychiatry, Psychology & Neuroscience, King’s College London, London, UK; 3 Department of Psychological Medicine, Institute of Psychiatry, Psychology & Neuroscience, King’s College London, London, UK

**Keywords:** Anxiety disorder, Bipolar disorder, Depression, Frailty, Mortality, UK Biobank

## Abstract

**Background:**

Frailty is a medical syndrome that is strongly associated with mortality risk and an emerging global health burden. Mental disorders are associated with reduced life expectancy and elevated levels of frailty. In this study, we examined the mortality risk associated with frailty in individuals with a lifetime history of mental disorders compared to individuals without a history of mental disorders.

**Methods:**

The UK Biobank study recruited > 500,000 adults, aged 37–73, between 2006 and 2010. We derived the two most common albeit distinctive measures of frailty, the frailty phenotype and the frailty index. Individuals with lifetime depression, bipolar disorder or anxiety disorders were identified from multiple data sources. The primary outcome was all-cause mortality. We have also examined differences in frailty, separately by sex and age.

**Results:**

Analyses included up to 297,380 middle-aged and older adults with a median follow-up of 12.19 (interquartile range = 1.31) years, yielding 3,516,706 person-years of follow-up. We observed higher levels of frailty in individuals with mental disorders for both frailty measures. Standardised mean differences in the frailty index ranged from 0.66 (95% confidence interval [CI] 0.65–0.67) in individuals with anxiety disorders to 0.94 (95% CI 0.90–0.97) in individuals with bipolar disorder, compared to people without mental disorders. For key comparisons, individuals with a mental disorder had greater all-cause mortality hazards than the comparison group without mental disorders. The highest hazard ratio (3.65, 95% CI 2.40–5.54) was observed among individuals with bipolar disorder and frailty, relative to non-frail individuals without mental disorders.

**Conclusions:**

Our findings highlight elevated levels of frailty across three common mental disorders. Frailty and mental disorders represent potentially modifiable targets for prevention and treatment to improve population health and life expectancy, especially where both conditions coexist.

**Supplementary Information:**

The online version contains supplementary material available at 10.1186/s12916-022-02474-2.

## Background

Frailty is a medical syndrome that is characterised by age-related declines in functioning across multiple physiological systems. Frail individuals have a decreased reserve capacity, leaving them less resilient to stressors and at an increased risk of adverse health outcomes. There are multiple approaches to operationalising frailty, most prominently Fried’s frailty phenotype [1] and the frailty index by Mitnitski, Mogilner and Rockwood [2]. The frailty phenotype comprises five specific indicators of physical capability, while the frailty index includes multiple health deficits across diverse physiological systems and can be adapted to routinely collected data. Frailty is strongly associated with mortality risk [3, 4] and represents an emerging global health burden [5]. The prevalence of frailty in community-dwelling older adults varies widely (range: 4.0% to 59.1%), with a weighted mean prevalence estimate of 10.7% in high-income countries [6]. Previous research showed that frailty was more common in females [6, 7] and in older adults [6].

Frailty is increasingly seen as a valuable clinical measure in psychiatric populations [8]. The estimated prevalence of frailty in older adults with depression is 40.4% (95% confidence interval: 27.0% to 55.3%) [9]. A recent systematic review found that the prevalence of frailty in individuals with severe mental illness varied from 10.2 to 89.7% [10]. Individuals with mental disorders are at an increased risk of physical comorbidities [11], have a lower life expectancy [12], differ from individuals without mental disorders in physiological markers [13–15] and may experience accelerated biological ageing [16, 17]. Frailty is associated with molecular indicators of ageing such as DNA methylation [18] and provides complementary information to other biomarkers [19, 20]. As such, it may be useful for risk stratification [21] and for predicting adverse health-related outcomes, including disability, falls, loss of independence and delayed recovery from illness. Frailty represents both a potential mechanism and synergistic factor contributing to the increased mortality risk of individuals with mental disorders.

However, few studies have investigated the mortality risk associated with frailty in adults with mental disorders [22]. As such, the primary aim of this study was to examine the mortality risk associated with frailty in individuals with a lifetime history of mental disorders. Using data from up to 297,380 participants in the UK Biobank, a major biomedical database, we examined all-cause mortality in individuals with depression, bipolar disorder and anxiety disorders. Frailty was assessed using two measures, the frailty phenotype and the frailty index, to enable distinctive yet complementary insights into the impact of frailty on mortality risk in mental disorders. Secondary aims of this study included examining cross-sectional differences in frailty between individuals with and without mental disorders, sex-specific effects and age-related differences in frailty.

## Methods

### Study population

The UK Biobank is a prospective study of more than 500,000 middle-aged and older adults (aged 37 to 73 years; target age range: 40 to 69 years), who were recruited between 2006 and 2010. The study rationale and design have been described elsewhere [23]. Briefly, individuals registered with the UK National Health Service (NHS) and living within a 25-mile (~40 km) radius of one of 22 assessment centres were invited to participate. Participants provided data on their sociodemographic characteristics, health behaviours and medical history and underwent physical examinations. Linked hospital inpatient records are available for most participants and primary care records are available for half of the participants. A third of the participants also completed an online follow-up mental health questionnaire (MHQ) between 2016 and 2017.

### Mental disorders

We identified individuals with a lifetime history of depression, bipolar disorder or anxiety disorders using criteria that we have reported elsewhere [13–15]. Cases were ascertained from multiple data sources: the modified Composite International Diagnostic Interview Short Form (CIDI-SF), self-report questions on (hypo)mania and a question on psychiatric diagnoses (UK Biobank data field 20544) which were assessed as part of the MHQ; the nurse-led baseline interview in which participants reported medical diagnoses (field 20002); hospital inpatient records (ICD-10 codes); primary care records (Read v2 or CTV3 codes) and self-report questions on mood disorders from the baseline assessment (field 20126). Individuals with psychosis were excluded from all cases and individuals with bipolar disorder were excluded from anxiety disorder cases due to their increased risk of physical multimorbidity [24, 25]. The depression and bipolar disorder groups were mutually exclusive, but individuals could be included in both the anxiety disorder and the depression group. Individuals could have had other psychiatric comorbidities (e.g. substance use or eating disorders), however these were not the focus of this study.

A non-psychiatric comparison group included individuals who had no mental disorders: (i) had not reported “schizophrenia”, “depression”, “mania / bipolar disorder / manic depression”, “anxiety / panic attacks”, “obsessive compulsive disorder”, “anorexia/bulimia/other eating disorder”, “post-traumatic stress disorder” at the nurse-led interview; (ii) reported no psychiatric diagnoses on the MHQ; (iii) reported no current psychotropic medication use at baseline (field 20003) [26]; (iv) had no ICD-10 Chapter V code in their hospital inpatient record, except for organic causes or substance use; (v) had no diagnostic codes for mental disorders in their primary care record [27]; (vi) were not classified as individuals with probable mood disorder at the baseline assessment; (vii) had no Patient Health Questionnaire-9 (PHQ-9) or Generalised Anxiety Disorder Assessment (GAD-7) sum score of ≥ 5; (viii) did not report that they ever felt worried, tense or anxious for most of a month or longer (field 20421); and (ix) were not identified as cases based on the CIDI-SF and questions on (hypo)mania [13, 15].

### Frailty phenotype

We derived the Fried frailty phenotype [1], adapted for the UK Biobank [28, 29]. Participants provided data on weight loss, exhaustion, physical activity and walking speed via touch-screen questionnaires at the baseline assessment (Additional file 1: Table S1). Hand-grip strength in whole kilogramme-force units was measured using a Jamar J00105 hydraulic hand dynamometer. We used the maximal grip strength of the participant’s self-reported dominant hand. If no data on handedness were available or the participant was ambidextrous, we used the highest value of both hands [30]. All variables were coded as zero or one and summed up. Participants with a total score of three or more were classified as frail, while participants with a total score of one or two and zero were classified as pre-frail and non-frail, respectively [1]. Participants with missing data for at least one criterion were excluded.

### Frailty index

We also derived a frailty index, following the procedure previously used in the UK Biobank [31]. Health deficits included in this index met the following criteria: indicators of poor health, more prevalent in older individuals, neither rare nor universal, covering multiple areas of human functioning and available for ≥ 80% of participants. The index included 49 variables obtained via touch-screen questionnaires and nurse-led interviews at the baseline assessment, including cardiometabolic, cranial, immunological, musculoskeletal, respiratory and sensory traits, well-being, infirmity, cancer and pain (Additional file 1: Table S2). Categorical variables were dichotomised (no deficit = 0; deficit = 1), and ordinal variables were mapped onto a score between zero and one. The sum of deficits present was divided by the total number of possible deficits, resulting in frailty index values between zero and one, with higher values reflecting greater levels of frailty [32, 33]. Participants with missing data for ≥ 10 variables were excluded [31]. Participants with a frailty index value of ≤ 0.08 were classified as non-frail, while participants with values between 0.08–0.25 and ≥ 0.25 were classified as pre-frail and frail, respectively [34].

### Ascertainment of mortality

The date of death was obtained through linkage with national death registries, NHS Digital (England and Wales) and the NHS Central Register (Scotland). The censoring date was 28 February 2021. The most recent death was recorded for 23 March 2021, although the data were incomplete for March 2021.

### Covariates

Covariates were identified from previous studies and included age, sex, ethnicity (White, Asian, Black, Chinese, Mixed-race or other) and highest educational/professional qualification (four levels, reflecting similar years of education [35]: college/university degree; education to age 18 or above, but not reaching degree level (“A levels”/“AS levels” or equivalent, NVQ/HND/HNC or equivalent, other professional qualifications); education to age 16 qualifications (“GCSEs”/“O levels” or equivalent, “CSEs” or equivalent; no qualifications), Townsend deprivation index, which is a small-area level measure of socioeconomic status [36], cohabitation with spouse or partner (yes/no) [37], smoking status (never, former or current), alcohol intake frequency (never, special occasions only, one to three times a month, once or twice a week, three or four times a week, or daily or almost daily), systolic and diastolic blood pressure (mmHg), body mass index (BMI, kg/m^2^), cholesterol (mmol/L), multimorbidity count (zero, one, two, three, four, five or more) and assessment centre.

### Statistical analyses

All statistical analyses and data visualisations were done in R (version 3.6.2).

Sample characteristics were summarised using means and standard deviations or counts and percentages. Differences in the frailty index between individuals with and without mental disorders were estimated using standardised mean differences ± 95% confidence intervals (CI) and ordinary least squares regression models. Group differences in the frailty phenotype (non-frail, pre-frail and frail) were estimated using ordinal logistic regression models. We fitted both unadjusted and fully adjusted models. Age-related differences in the frailty index were estimated using generalised additive models within the ‘mgcv’ package [38] in R.

We calculated person-years of follow-up and the median duration of follow-up of censored individuals. Unadjusted survival probabilities by frailty level and case status were estimated using the Kaplan-Meier (KM) method [39]. Hazard ratios (HRs) and 95% confidence intervals were estimated using Cox proportional hazards models [40] to examine associations between frailty and mortality by case status. Age in years was used as the underlying time axis, with age 40 as the start of follow-up. We fitted both unadjusted and fully adjusted models. Non-frail individuals without mental disorders were the reference group. We estimated the percentage risk difference between individuals with and without mental disorders at the pre-frailty and frailty levels using the formula: (HR_disorder_ – HR_no disorder_)/(HR_no disorder_ − 1) × 100.

Adjusted *P*-values were calculated using the *p.adjust* function in R to account for multiple testing. *P*-values from the regression models were corrected for six tests (one parameter × two models × three disorders) and *p*-values from the Cox proportional hazards models for 30 tests (five parameters × two models × three disorders). Two methods were used: (1) Bonferroni and (2) Benjamini and Hochberg [41], two-tailed, with *α* = .05 and a 5% false discovery rate, respectively. We have opted for this approach because the Bonferroni correction may be too conservative and lead to a high number of false negatives.

### Additional analyses

We repeated our main analyses of group differences in frailty and of all-cause mortality stratified by sex. As a sensitivity analysis, we repeated the analyses of all-cause mortality after excluding individuals with comorbid depression and anxiety disorders. Finally, we examined all-cause mortality in individuals with comorbid depression and anxiety disorders.

## Results

The analytical samples included up to 297,380 participants. 76,586 individuals had a lifetime history of depression, 3029 individuals had bipolar disorder and 37,779 individuals had lifetime anxiety disorders. The non-psychiatric comparison group included 220,794 participants. The percentage of individuals with frailty based on the frailty phenotype ranged from 1.8% in the non-psychiatric comparison group to 5.5% in individuals with bipolar disorder. The sample characteristics are presented in Table 1. There was moderate overlap between the frailty phenotype and the frailty index categories (Additional file 1: Fig. S1).

**Table 1:** Sample characteristics of individuals with and without mental disorders

	Depression	Bipolar disorder	Anxiety disorder	No mental disorder
	*N*=76586	*N*=3029	*N*=37779	*N*=220794
**Age**				
Mean (SD)	55.13 (7.92)	54.31 (8.00)	55.72 (7.89)	56.43 (8.15)
**Sex**				
Female	50040 (65.3%)	1710 (56.5%)	24944 (66.0%)	109447 (49.6%)
Male	26546 (34.7%)	1319 (43.5%)	12835 (34.0%)	111347 (50.4%)
**Neighbourhood deprivation**				
Mean (SD)	− 1.48 (2.91)	− 1.10 (3.02)	− 1.62 (2.87)	− 1.78 (2.83)
**Ethnicity**				
White	73868 (96.5%)	2855 (94.3%)	36754 (97.3%)	209251 (94.8%)
Mixed-race	522 (0.7%)	28 (0.9%)	191 (0.5%)	1105 (0.5%)
Black	613 (0.8%)	41 (1.4%)	248 (0.7%)	3280 (1.5%)
Asian	955 (1.2%)	67 (2.2%)	351 (0.9%)	4521 (2.0%)
Chinese	120 (0.2%)	6 (0.2%)	42 (0.1%)	822 (0.4%)
Other	508 (0.7%)	32 (1.1%)	193 (0.5%)	1815 (0.8%)
**Highest qualification**				
None	10642 (13.9%)	316 (10.4%)	5197 (13.8%)	35669 (16.2%)
O levels/GCSEs/CSEs	21309 (27.8%)	794 (26.2%)	10465 (27.7%)	61512 (27.9%)
A levels/NVQ/HND/HNC^a^	18105 (23.6%)	714 (23.6%)	8931 (23.6%)	51268 (23.2%)
Degree	26530 (34.6%)	1205 (39.8%)	13186 (34.9%)	72345 (32.8%)
**Spouse/partner cohabitation**				
No	10864 (14.2%)	521 (17.2%)	4736 (12.5%)	19148 (8.7%)
Yes	65722 (85.8%)	2508 (82.8%)	33043 (87.5%)	201646 (91.3%)
**Smoking status**				
Never	39622 (51.7%)	1408 (46.5%)	19868 (52.6%)	126857 (57.5%)
Former	28321 (37.0%)	1122 (37.0%)	14128 (37.4%)	75365 (34.1%)
Current	8643 (11.3%)	499 (16.5%)	3783 (10.0%)	18572 (8.4%)
**Alcohol intake frequency**				
Never	6242 (8.2%)	329 (10.9%)	3163 (8.4%)	14311 (6.5%)
Special occasions	9487 (12.4%)	410 (13.5%)	4552 (12.0%)	21354 (9.7%)
1–3/month	9314 (12.2%)	352 (11.6%)	4256 (11.3%)	22680 (10.3%)
1–2/week	19101 (24.9%)	724 (23.9%)	9271 (24.5%)	59197 (26.8%)
3–4/week	16966 (22.2%)	578 (19.1%)	8554 (22.6%)	55923 (25.3%)
Daily/almost daily	15476 (20.2%)	636 (21.0%)	7983 (21.1%)	47329 (21.4%)
**Body mass index**				
Mean (SD)	27.68 (5.07)	28.15 (5.37)	27.36 (4.96)	27.19 (4.44)
**Systolic blood pressure**				
Mean (SD)	135.00 (18.15)	133.79 (17.61)	135.89 (18.34)	138.64 (18.61)
**Diastolic blood pressure**				
Mean (SD)	81.54 (10.06)	81.53 (10.15)	81.66 (10.02)	82.50 (10.06)
**Cholesterol**				
Mean (SD)	5.72 (1.14)	5.67 (1.15)	5.74 (1.13)	5.70 (1.13)
**Multimorbidity count**				
None	12689 (16.6%)	380 (12.5%)	5930 (15.7%)	62516 (28.3%)
One	18249 (23.8%)	651 (21.5%)	8790 (23.3%)	62660 (28.4%)
Two	15760 (20.6%)	620 (20.5%)	7939 (21.0%)	43558 (19.7%)
Three	11656 (15.2%)	516 (17.0%)	5934 (15.7%)	25471 (11.5%)
Four	7485 (9.8%)	342 (11.3%)	3784 (10.0%)	13374 (6.1%)
Five or more	10747 (14.0%)	520 (17.2%)	5402 (14.3%)	13215 (6.0%)
**Antidepressant use**				
No	60326 (78.8%)	2175 (71.8%)	29869 (79.1%)	220794 (100.0%)
Yes	16260 (21.2%)	854 (28.2%)	7910 (20.9%)	0 (0.0%)
**Antipsychotic use**				
No	76276 (99.6%)	2827 (93.3%)	37586 (99.5%)	220794 (100.0%)
Yes	310 (0.4%)	202 (6.7%)	193 (0.5%)	0 (0.0%)
**Lithium use**				
No	76486 (99.9%)	2755 (91.0%)	37740 (99.9%)	220794 (100.0%)
Yes	100 (0.1%)	274 (9.0%)	39 (0.1%)	0 (0.0%)
**Frailty phenotype**				
Non-frail	35473 (46.3%)	1284 (42.4%)	18007 (47.7%)	125980 (57.1%)
Pre-frail	37549 (49.0%)	1579 (52.1%)	18199 (48.2%)	90881 (41.2%)
Frail	3564 (4.7%)	166 (5.5%)	1573 (4.2%)	3933 (1.8%)
**Frailty phenotype count**				
None	35473 (46.3%)	1284 (42.4%)	18007 (47.7%)	125980 (57.1%)
One	27560 (36.0%)	1143 (37.7%)	13578 (35.9%)	72679 (32.9%)
Two	9989 (13.0%)	436 (14.4%)	4621 (12.2%)	18202 (8.2%)
Three	2870 (3.7%)	134 (4.4%)	1264 (3.3%)	3376 (1.5%)
Four	632 (0.8%)	30 (1.0%)	277 (0.7%)	523 (0.2%)
Five	62 (0.1%)	2 (0.1%)	32 (0.1%)	34 (0.0%)
Mean (SD)	0.77 (0.88)	0.84 (0.91)	0.74 (0.86)	0.55 (0.73)
**Frailty index**				
Mean (SD)	0.15 (0.08)	0.17 (0.08)	0.15 (0.08)	0.10 (0.06)
**Frailty index categories**				
Non-frail	14397 (18.8%)	445 (14.7%)	6641 (17.6%)	88189 (39.9%)
Pre-frail	53280 (69.6%)	2098 (69.3%)	26581 (70.4%)	125648 (56.9%)
Frail	8909 (11.6%)	486 (16.0%)	4557 (12.1%)	6957 (3.2%)

*SD* standard deviation, *GCSEs* general certificate of secondary education, *CSE* certificate of secondary education, *NVQ* national vocational qualification, *HND* higher national diploma, *HNC* higher national certificate

^a^Also includes ‘other professional qualifications’

Cut-offs for frailty index categories were non frail (≤ 0.08), pre-frail (> 0.08 and < 0.25) and frail (≥ 0.25)

**Fig. 1:**
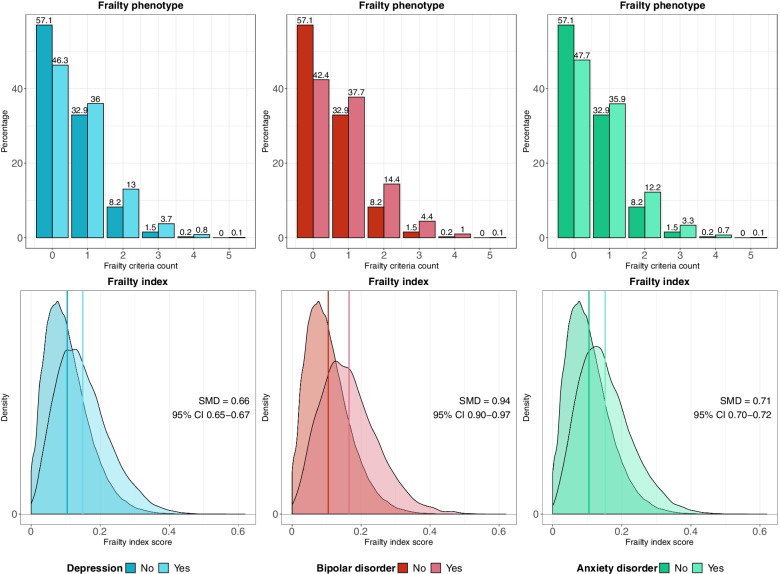
Histograms and density plots showing the distribution of the frailty phenotype criteria count (top panels) and the frailty index (bottom panels), respectively, for individuals with and without mental disorders

For the frailty phenotype, the percentage of participants with pre-frailty or frailty was higher in individuals with mental disorders than in the non-psychiatric comparison group (Table 1). We observed a similar pattern for the frailty criteria count, showing that the percentage of individuals with mental disorders was higher than the percentage of individuals without mental disorders for scores one to five (Fig. 1). The largest difference was observed between individuals with bipolar disorder and the non-psychiatric comparison group.

**Fig. 2:**
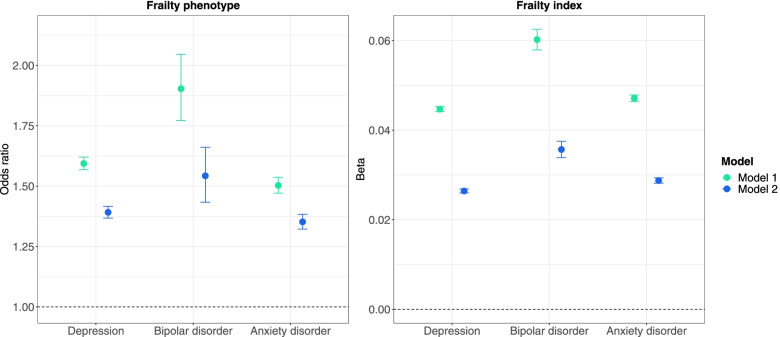
Frailty in individuals with mental disorders compared to individuals without mental disorders (reference group). Estimates shown for the frailty phenotype are odds ratios and 95% confidence intervals (CI) from ordinal logistic regression models, indicating changes in odds of being frailer associated with being in the case group relative to the comparison group without mental disorders. Estimates shown for the frailty index are ordinary least squares regression beta coefficients and 95% CI. Model 1—unadjusted; Model 2—adjusted for age, sex, ethnicity, highest qualification, Townsend deprivation index, cohabitation with spouse/partner, smoking status, alcohol intake frequency, systolic and diastolic blood pressure, body mass index, cholesterol, multimorbidity count and assessment centre

Frailty index scores were also higher in individuals with mental disorders, with the largest standardised mean difference (SMD) observed between individuals with bipolar disorder and the non-psychiatric comparison group (SMD = 0.94, 95% CI 0.90–0.97, *p* < 0.001) (Fig. 1). Individuals with mental disorders also had higher levels of frailty after adjustment for potential confounders in regression models, irrespective of how frailty was operationalized (Fig. 2 and Table 2).

**Table 2:** Frailty in individuals with and without mental disorders

	Model 1			Model 2		
**Frailty phenotype**	OR	95% CI		OR	95% CI	
No mental disorder	Ref	–	–	Ref	–	–
Depression	1.594	1.568	1.620	1.392	1.368	1.416
Bipolar disorder	1.904	1.772	2.046	1.543	1.433	1.661
Anxiety disorder	1.503	1.471	1.536	1.352	1.322	1.384
**Frailty index**	*β*	95% CI		*β*	95% CI	
No mental disorder	Ref	–	–	Ref	–	–
Depression	0.045	0.044	0.045	0.026	0.026	0.027
Bipolar disorder	0.060	0.058	0.063	0.036	0.034	0.038
Anxiety disorder	0.047	0.046	0.048	0.029	0.028	0.029

*OR* odds ratio, *β* ordinary least squares regression beta coefficient, *CI* confidence interval, *Ref* reference group. All Bonferroni-adjusted *p*-values < 0.001. Odds ratios indicate changes in odds of being frailer associated with being in the case group relative to the comparison group without mental disorders. Model 1—unadjusted; Model 2—adjusted for age, sex, ethnicity, highest qualification, Townsend deprivation index, cohabitation with spouse/partner, smoking status, alcohol intake frequency, systolic and diastolic blood pressure, body mass index, cholesterol, multimorbidity count and assessment centre

Sample characteristics stratified by sex are presented in Additional file 1: Table S3. For the frailty phenotype, females with mental disorders had higher levels of pre-frailty and frailty than males. A similar pattern emerged with respect to the frailty criteria count, although few individuals had a score of five (Additional file 1: Fig. S2). Frailty index scores were higher among females compared to males with anxiety disorders, but the magnitude of this difference was negligible compared to the differences between individuals with mental disorders and the non-psychiatric comparison group. There was no evidence of a difference in frailty index scores between males or females with depression or bipolar disorder and the non-psychiatric comparison group (Additional file 1: Table S4). In the sex-stratified regression models, both males and females with mental disorders had higher levels of frailty than the non-psychiatric comparison group, including after adjustment for potential confounders (Additional file 1: Fig. S3 and Table S5). For the frailty phenotype, estimates for females were slightly higher than for males, relative to the comparison group, while we observed mostly the reverse pattern for the frailty index. However, the magnitude of these differences in estimates was negligible.

**Fig. 3:**
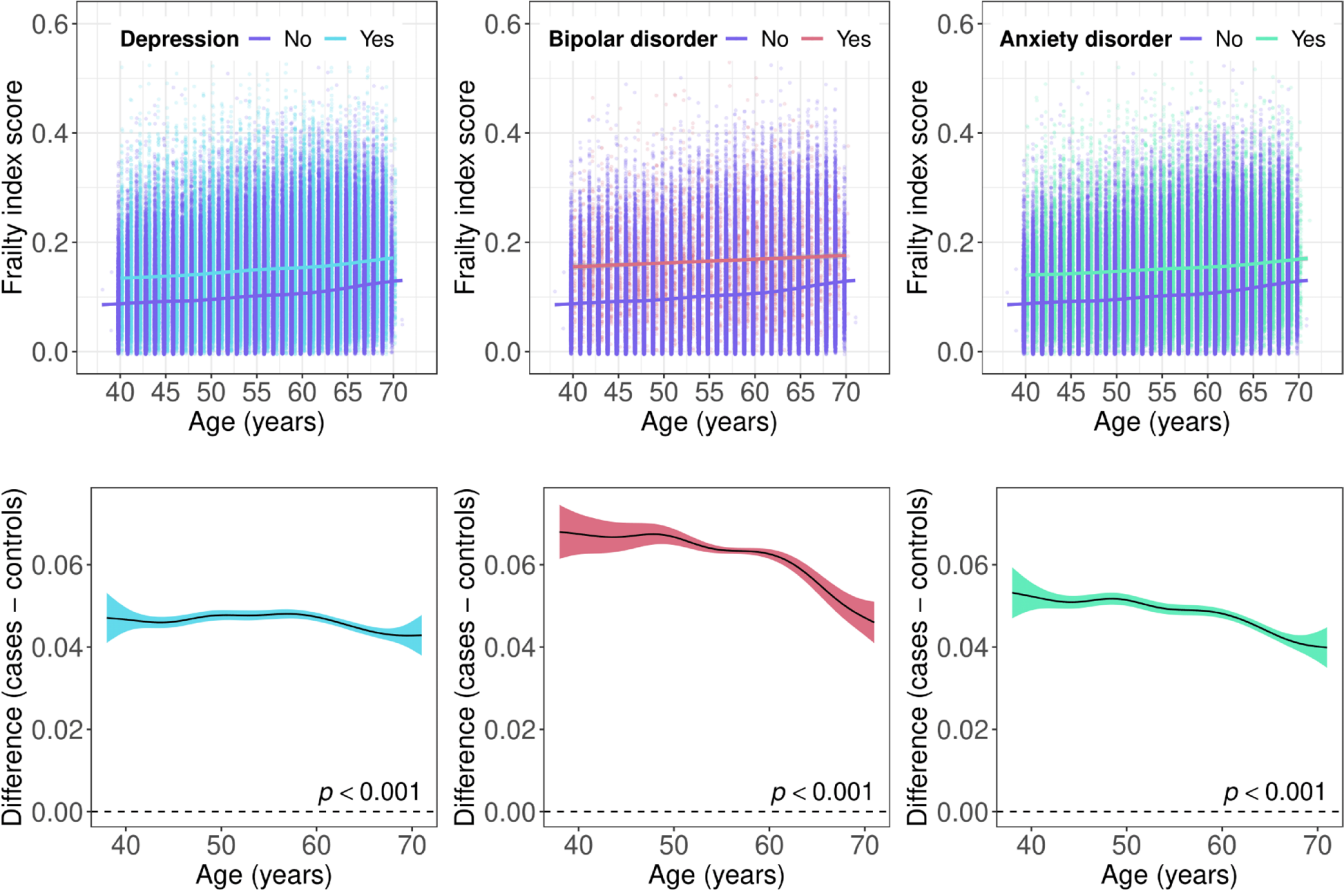
Top panels: scatter plots showing the frailty index by age in individuals with and without mental disorders. Bottom panels: difference smooths comparing age-related differences in frailty index scores of individuals with and without mental disorders. Positive values on the *y*-axes correspond to higher frailty index scores in individuals with mental disorders. The smooth curves were estimated using generalised additive models. The shaded areas correspond to 95% confidence intervals

Frailty index scores increased with age in individuals with mental disorders and in the non-psychiatric comparison group. We found some evidence that the group differences in frailty between individuals with and without mental disorders narrowed above age 60, resulting from a steeper age-related increase in frailty in individuals without mental disorders (Fig. 3).

Differences in the frailty phenotype between individuals with and without mental disorders were fairly consistent across the age spectrum. The combined percentage of participants with pre-frailty or frailty was greater in individuals with mental disorders at most ages, with median estimates of 53.72% in depression, 57.14% in bipolar disorder, 53.65% in anxiety disorders and 43.37% in the non-psychiatric comparison group (Additional file 1: Fig. S4).

### All-cause mortality

The median duration of follow-up of censored individuals was between 12.09 (IQR = 1.35) and 12.1 (IQR = 1.31) years, with 2,654,566 to 3,516,706 person-years of follow-up (Additional file 1: Table S6). Individuals with depression or bipolar disorder had a greater all-cause mortality hazard than individuals without mental disorders, while we did not observe an increased mortality risk in individuals with anxiety disorders (Additional file 1: Fig. S5 and Table S7). Regardless of frailty measure, the hazards for all-cause mortality were greater among pre-frail and frail participants (Additional file 1: Fig. S6 and Table S8). Survival probabilities by frailty level and case status are presented in Additional file 1: Figs. S7 and S8.

Considering the frailty phenotype, the largest hazard ratio (HR) was observed for individuals with bipolar disorder and frailty (HR = 3.65, 95% CI 2.40–5.54) compared to non-frail individuals without mental disorders (Table 3). A similar pattern of results was revealed for depression and anxiety disorders, except that pre-frail individuals with anxiety disorders had a lower hazard ratio than pre-frail individuals without mental disorders. Further, the differences in all-cause mortality were not statistically significant between non-frail individuals with depression or anxiety disorders and non-frail individuals without mental disorders. Adjustment for potential confounders attenuated the effect sizes, but the differences persisted (Additional file 1: Fig. S9). The results from the Cox proportional hazards models in which we examined the frailty index categories suggested further differences from the analysis of the frailty phenotype (Table 4). For instance, several estimates suggested lower all-cause mortality hazards in individuals with depression or anxiety disorders, relative to individuals without mental disorders, both in the pre-frail and frail groups (Additional file 1: Fig. S10).

**Table 3:** All-cause mortality by frailty phenotype in individuals with and without mental disorders

		Model 1					RD %	Model 2					RD %
		HR	95% CI		*p*_Bonf._	*p*_BH_		HR	95% CI		*p*_Bonf._	*p*_BH_	
**Depression**													
Non-frail	No	Ref	–	–	–	–	–	Ref	–	–	–	–	–
Non-frail	Yes	1.042	0.986	1.101	>0.999	0.158	–	1.016	0.960	1.074	>0.999	0.590	–
Pre-frail	No	1.352	1.305	1.400	<0.001	<0.001	–	1.233	1.190	1.278	<0.001	<0.001	–
Pre-frail	Yes	1.457	1.387	1.530	<0.001	<0.001	29.95	1.263	1.200	1.329	<0.001	<0.001	12.91
Frail	No	2.864	2.630	3.118	<0.001	<0.001	–	1.968	1.803	2.149	<0.001	<0.001	–
Frail	Yes	3.289	2.990	3.619	<0.001	<0.001	22.83	2.104	1.905	2.325	<0.001	<0.001	14.02
**Bipolar disorder**													
Non-frail	No	Ref	–	–	–	–	–	Ref	–	–	–	–	–
Non-frail	Yes	1.602	1.276	2.011	0.002	<0.001	–	1.359	1.082	1.707	0.253	0.010	–
Pre-frail	No	1.352	1.305	1.400	<0.001	<0.001	–	1.232	1.188	1.276	<0.001	<0.001	–
Pre-frail	Yes	2.144	1.784	2.576	<0.001	<0.001	225.04	1.681	1.397	2.022	<0.001	<0.001	193.89
Frail	No	2.864	2.631	3.118	<0.001	<0.001	–	1.959	1.792	2.142	<0.001	<0.001	–
Frail	Yes	3.647	2.400	5.543	<0.001	<0.001	42.01	2.296	1.508	3.495	0.003	<0.001	35.06
**Anxiety disorder**													
Non-frail	No	Ref	–	–	–	–	–	Ref	–	–	–	–	–
Non-frail	Yes	0.976	0.907	1.049	>0.999	0.520	–	0.972	0.903	1.046	>0.999	0.478	–
Pre-frail	No	1.352	1.305	1.400	<0.001	<0.001	–	1.232	1.189	1.276	<0.001	<0.001	–
Pre-frail	Yes	1.212	1.132	1.298	<0.001	<0.001	− 39.79	1.089	1.016	1.169	0.493	0.019	− 61.39
Frail	No	2.864	2.631	3.118	<0.001	<0.001	–	1.958	1.792	2.139	<0.001	<0.001	–
Frail	Yes	3.031	2.625	3.500	<0.001	<0.001	8.97	2.041	1.762	2.364	<0.001	<0.001	8.69

*HR* hazard ratio, *CI* confidence interval, *RD %* percentage risk difference, *Ref* reference group, *Bonf.* Bonferroni, *BH* Benjamini and Hochberg. Age (in years) was used as the underlying time axis. Model 1—unadjusted; Model 2—adjusted for sex, ethnicity, highest qualification, Townsend deprivation index, cohabitation with spouse/partner, smoking status, alcohol intake frequency, systolic and diastolic blood pressure, body mass index, cholesterol, multimorbidity count and assessment centre

**Table 4:** All-cause mortality by frailty index categories in individuals with and without mental disorders

		Model 1						Model 2					
		HR	95% CI		*p*_Bonf._	*p*_BH_	RD %	HR	95% CI		*p*_Bonf._	*p*_BH_	RD %
**Depression**													
Non-frail	No	Ref	–	–	–	–	–	Ref	–	–	–	–	–
Non-frail	Yes	1.050	0.954	1.157	>0.999	0.354	–	1.081	0.981	1.191	>0.999	0.137	–
Pre-frail	No	1.390	1.336	1.446	<0.001	<0.001	–	1.168	1.118	1.221	<0.001	<0.001	–
Pre-frail	Yes	1.372	1.305	1.443	<0.001	<0.001	− 4.64	1.174	1.111	1.241	<0.001	<0.001	3.53
Frail	No	2.564	2.393	2.746	<0.001	<0.001	–	1.622	1.498	1.755	<0.001	<0.001	–
Frail	Yes	2.555	2.380	2.744	<0.001	<0.001	− 0.55	1.606	1.479	1.744	<0.001	<0.001	− 2.48
**Bipolar disorder**													
Non-frail	No	Ref	–	–	–	–	–	Ref	–	–	–	–	–
Non-frail	Yes	1.297	0.805	2.089	>0.999	0.329	–	1.214	0.754	1.956	>0.999	0.455	–
Pre-frail	No	1.389	1.335	1.445	<0.001	<0.001	–	1.164	1.113	1.218	<0.001	<0.001	–
Pre-frail	Yes	2.034	1.715	2.411	<0.001	<0.001	165.90	1.540	1.296	1.831	<0.001	<0.001	229.15
Frail	No	2.559	2.389	2.742	<0.001	<0.001	–	1.604	1.478	1.742	<0.001	<0.001	–
Frail	Yes	3.714	2.865	4.814	<0.001	<0.001	74.02	2.220	1.704	2.891	<0.001	<0.001	101.77
**Anxiety disorder**													
Non-frail	No	Ref	–	–	–	–	–	Ref	–	–	–	–	–
Non-frail	Yes	1.002	0.877	1.144	>0.999	0.981	–	1.043	0.913	1.191	>0.999	0.552	–
Pre-frail	No	1.389	1.335	1.445	<0.001	<0.001	–	1.169	1.118	1.222	<0.001	<0.001	–
Pre-frail	Yes	1.198	1.124	1.277	<0.001	<0.001	− 49.13	1.063	0.993	1.138	>0.999	0.109	− 62.95
Frail	No	2.561	2.390	2.744	<0.001	<0.001	–	1.604	1.480	1.739	<0.001	<0.001	–
Frail	Yes	2.107	1.905	2.331	<0.001	<0.001	− 29.06	1.403	1.258	1.566	<0.001	<0.001	− 33.27

*HR* hazard ratio, *CI* confidence interval, *RD %* percentage risk difference, *Ref* reference group, *Bonf.* Bonferroni, *BH* Benjamini and Hochberg. Age (in years) was used as the underlying time axis. Model 1—unadjusted; Model 2—adjusted for sex, ethnicity, highest qualification, Townsend deprivation index, cohabitation with spouse/partner, smoking status, alcohol intake frequency, systolic and diastolic blood pressure, body mass index, cholesterol, multimorbidity count and assessment centre

### Additional analyses

The results of the analyses of the frailty phenotype and all-cause mortality stratified by sex are presented in Additional file 1: Fig. S11 and Table S9. Overall, males presented with modestly greater all-cause mortality hazards relative to females. Compared to the non-frail group without mental disorders, pre-frail females with depression had a higher risk of all-cause mortality (though the effect size in the adjusted model was lower relative to the pre-frail females without mental disorders). Frail males with bipolar disorder had a greater all-cause mortality hazard compared to females (adjusted HR = 3.11, 95% CI 1.87–5.18 and HR = 1.39, 95% CI 0.66–2.92, respectively), relative to non-frail individuals without mental disorders, while the reverse was observed for the pre-frail groups. Moreover, frail females with anxiety disorders presented with higher all-cause mortality hazards than their male counterparts, relative to the non-frail individuals without mental disorders. Of note, frail males with anxiety disorders had a lower risk of all-cause mortality relative to their counterparts without mental disorders (adjusted HR = 1.87, 95% CI 1.48–2.35 and HR = 2.01, 95% CI 1.79–2.26, respectively). The overall pattern of results from the sex-stratified models of the frailty index categories was comparable to the results from the main analysis (Additional file 1: Fig. S12 and Table S10). Pre-frail females with bipolar disorder, anxiety disorders or without mental disorders had a higher mortality hazard than males, relative to non-frail individuals without mental disorders. The mortality hazard of non-frail females with bipolar disorder was also higher than in males.

The results for the subset of participants from which we excluded individuals with comorbid depression and anxiety disorders (*n* = 23,712) are shown in Additional file 1: Tables S11 to S13. Overall, the all-cause mortality hazards were slightly elevated in these analyses. In the analyses in which we examined individuals with comorbid depression and anxiety disorders, we found that the all-cause mortality hazards were slightly attenuated (Additional file 1: Tables S9 to S11). Notably, these sensitivity analyses showed that individuals with depression and no comorbid anxiety disorder had an increased all-cause mortality hazard relative to individuals without mental disorders at both the pre-frailty and frailty level.

## Discussion

We observed higher levels of frailty in individuals with mental disorders compared to people without mental disorders, regardless of how frailty was operationalized. The frailty phenotype measure was consistent in documenting higher pre-frailty and frailty levels in females compared to males with mental disorders. Evidence for sex differences in the frailty index was observed mainly within anxiety disorders, with females demonstrating higher frailty scores relative to males. Notably, our findings suggested that differences in the frailty index scores between individuals with mental disorders and a non-psychiatric comparison group narrowed above age 60. On the other hand, there were mostly consistent differences in the frailty phenotype (both at the pre-frail and frail levels) between individuals with and without mental disorders across the age spectrum.

The above differences in frailty levels translated into an increased risk of all-cause mortality among individuals with a lifetime history of depression or bipolar disorders with respect to both the frailty phenotype and the frailty index measures. The association between frailty and anxiety disorders or comorbid depression and anxiety disorders with all-cause mortality was less consistent, with some evidence of lower mortality risk in these groups compared to the non-psychiatric comparison group. Possible explanations for this finding, if replicated, should be explored in further studies. Concerning sex differences, our findings revealed increased all-cause mortality among males relative to females, with certain exceptions. For instance, pre-frail males with bipolar disorder and frail males with anxiety disorders appeared to have a lower risk of all-cause mortality relative to females.

Previous studies that have examined differences in frailty between individuals with and without mental disorders have focussed on depression and older adults [9, 42]. A meta-analysis of 24 cross-sectional and longitudinal studies suggested increased frailty levels among people with depression [9]. Our study findings supported this evidence and extended it to individuals with bipolar disorder or anxiety disorders. These results are consistent with a large body of evidence indicating that individuals with mental disorders have poor physical health, an increased prevalence of medical comorbidities and a lower life expectancy relative to people without mental disorders [11, 12, 43]. Our finding that differences in the frailty index between individuals with and without mental disorders narrowed with age is consistent with a previous study showing that the relationship between depressive symptoms and the frailty phenotype weakened as people aged [44]. A potential explanation for this could be better coping strategies in older individuals. The lack of evidence of age-related increases in the frailty phenotype may be due to the younger age of the participants in our study, as previous research in adults aged 65 years or older observed increased frailty at older ages [6]. It could also be due to selection bias resulting in healthier older adults participating at greater rates, although we did observe higher frailty index scores in older participants. Recently, we have observed a decline in the prevalence of common mental disorders among adults in their late 50s to early 60s, followed by a sharp increase afterwards [45]. This trend represents another possible explanation for the decline in the strength of the association between the frailty phenotype and mental disorders in individuals over 60 years of age observed in the current study. The observation that frailty was increased in females whereas all-cause mortality was elevated in males is consistent with the male-female health-mortality paradox that has been documented elsewhere [7, 46]. Although biological, behavioural, and social factors have been hypothesised to explain this finding, the evidence for possible explanations (e.g. increased health-care use by females) has generally not been conclusive [46].

The dose-response association between the frailty phenotype levels and mortality that we observed in this study is consistent with a meta-analysis of population-based studies that included > 35,000 adults aged 65 years and above [3]. However, there has been little research to date examining the mortality risk associated with frailty in individuals with specific mental disorders. Findings from a prospective study of 2565 men aged 75 years or older suggested that current symptoms of depression, but not lifetime depression, were associated with increased all-cause mortality and that this association was largely due to differences in frailty [47]. Another recent study (*N* = 378) of patients with depression aged 60 years or older documented that the frailty phenotype count was associated with increased mortality risk [22]. Another study (*N* = 120) with older adults who were admitted for psychiatric inpatient treatment suggested that frailty was a strong predictor of mortality within this population [48]. However, this study did not provide data on disorder-specific mortality rates associated with frailty. Finally, a previous study of multimorbidity and frailty suggested that individuals with neuropsychiatric multimorbidity had the highest mortality rate for each level of frailty [49]. To the best of our knowledge, our study is the first to examine the mortality risk associated with frailty in individuals with bipolar disorder or anxiety disorders. The observation that the mortality risk was greatest in individuals with bipolar disorder is consistent with previous studies showing that bipolar disorder was associated with a greater mortality risk than depression or anxiety disorders [50, 51]. This could be due to people with bipolar disorder having higher rates of suicide attempts and death relative to those with depression or anxiety disorders. Further, bipolar disorder is associated with poor management of co-existent physical disorders [11, 52] that may also account for the higher mortality observed in this condition relative to depression or anxiety disorders in our study.

The inclusion of distinctive indicators of frailty enabled us to provide more robust evidence about the role of frailty in premature mortality among a large group of people with mental disorders. The two frailty measures tend to focus on different aspects of physical health, which may explain some of the discrepancies observed in our study. The frailty index has superior ability to discriminate at the lower to the middle end distribution of the frailty continuum [53], hence the lower hazard ratios for mortality compared to the frailty phenotype observed in our study. This suggestion is supported by the higher proportion of patients identified as frail with the frailty index relative to the frailty phenotype measure. The identification of subpopulations at risk of accelerated physiological decline is informative for the implementation of preventative strategies aimed at reducing the excess mortality in individuals with mental disorders. In general, physical frailty arises from dysregulation in multiple and dynamic body systems over long periods of time [54]. Individually tailored multicomponent interventions (e.g. physical activity, diet, psycho-social support and integrated care models) are likely to offer the best prognosis for ameliorating frailty within mental health populations. The evidence for the efficacy of such interventions to modify frailty in individuals with mental disorders is currently limited. In the meantime, the focus should be on minimising potentially aggravating factors for frailty in people with mental disorders, such as inappropriate polypharmacy, lack of care continuity or social isolation, while optimising integrated care and healthy behaviours (e.g. physical activity) [55]. Further longitudinal studies are needed to understand how the progression of frailty impacts on the progression of mental disorders and vice versa. In addition, further studies using multisystem physiological markers of frailty may help detect the inflection point for frailty-related mortality in people with mental disorders. Since the frailty index is in part operationalized by the presence of diagnosed physical comorbidity, it might be more suitable for older individuals. Its clinically utility for prevention of occurrence of physical morbidity may be limited in individuals with mental disorders who usually experience their illness onset in late adolescence or early adulthood. Future studies assessing the clinical utility of frailty compared to traditional risk factors for specific disease outcomes (e.g. cardiovascular disease mortality) [56] should be conducted in people with mental disorders.

A strength of this study is the large sample size of almost 300,000 participants with a median follow-up of 12 years. We focussed on two distinctive yet complementary measures of frailty, the frailty phenotype and frailty index and examined their association with all-cause mortality among three mental disorders with considerable disease burden. While research on frailty has predominantly been conducted in individuals aged 65 years and above, our study sample included both middle-aged and older adults, highlighting the association of frailty with all-cause mortality at the transition from middle age to late adulthood years.

Our observational research has certain limitations. As we have discussed elsewhere [57, 58], UK Biobank participants are healthier than the UK general population. As such, individuals with high levels of frailty and with chronic and/or severe mental disorders may have been less likely to be included in our study. This could have resulted in attenuated differences in frailty between individuals with and without mental disorders and reduced the corresponding mortality risk. For a discussion of the potential limitations regarding the ascertainment of individuals with mental disorders in the UK Biobank, see our previous studies [13–15]. There is conceptual overlap between frailty and some of the symptoms that characterise mental disorders, which could partially explain differences in frailty observed between individuals with and without mental disorders. However, a previous study of community-dwelling older adults showed that shared symptoms explained only part of the association between depression and both the frailty index and phenotype [59]. There is also overlap between the concept of frailty and physical multimorbidity. Given the evidence that individuals with mental disorders have a higher prevalence of physical multimorbidity [24, 25], this likely also contributes to differences in frailty observed between individuals with and without mental disorders. Nevertheless, multimorbidity is not synonymous with frailty [60] and we observed differences in frailty also after adjusting for a multimorbidity count. Our study provides limited insight into the causal relationships between mental disorders and frailty in relation to mortality. However, it is likely that mental illness and frailty are mutually reinforcing and may share common risk factors [61]. Finally, we cannot exclude the possibility of residual confounding, and other variables (e.g. genetics, healthcare access, drug prescriptions or global cognitive function) not considered here may also affect the observed associations. Our estimation models did adjust for a wide range of known confounders, however, minimising the potential risk from residual bias.

## Conclusion

Our findings highlight elevated levels of frailty across three common mental disorders. Screening for frailty might help identify individuals with mental disorders who are at risk of premature mortality. Screening for poor mental health is equally important as mental disorders tend to be under-recognised in individuals presenting with high levels of frailty and physical comorbidities. There is increasing evidence that frailty can be prevented, treated and potentially delayed. Frailty and mental disorders represent potentially modifiable targets for prevention and treatment to improve population health and life expectancy, especially where frailty and mental disorders coexist.

## Supplementary Information

**Additional file 1: **Full criteria used to identify individuals with mental disorders. **Table S1.** Frailty phenotype criteria. **Table S2.** Frailty index variables. **Figure S1.** Overlap between frailty phenotype and frailty index categories. **Table S3.** Sample characteristics stratified by sex. **Figure S2.** Frailty group differences stratified by sex. **Table S4.** Sex differences in frailty index. **Figure S3.** Sex-stratified regression models. **Table S5.** Sex-stratified regression models. **Figure S4.** Frailty phenotype by age. **Table S6.** Descriptive statistics follow-up. **Figure S5.** All-cause mortality by case status. **Table S7.** All-cause mortality by case status. **Figure S6.** All-cause mortality by frailty level. **Table S8.** All-cause mortality by frailty level. **Figure S7.** Survival probabilities by frailty phenotype and case status. **Figure S8.** Survival probabilities by frailty index categories and case status. **Figure S9.** All-cause mortality by frailty phenotype and case status. **Figure S10.** All-cause mortality by frailty index categories and case status. **Figure S11.** All-cause mortality by frailty phenotype and case status stratified by sex. **Table S9.** All-cause mortality by frailty phenotype and case status stratified by sex. **Figure S12.** All-cause mortality by frailty index categories and case status stratified by sex. **Table S10.** All-cause mortality by frailty index categories and case status stratified by sex. **Table S11.** Descriptive statistics follow-up, comorbidity depression and anxiety disorders. **Table S12.** All-cause mortality by frailty phenotype, comorbidity depression and anxiety disorders. **Table S13.** All-cause mortality by frailty index categories, comorbidity depression and anxiety disorders.

## Data Availability

The data used in the present study are available to all bona fide researchers for health-related research that is in the public interest, subject to an application process and approval criteria. Study materials are publicly available online at http://www.ukbiobank.ac.uk.
