# Supplementary material for: Frailty in individuals with depression, bipolar disorder and anxiety disorders: longitudinal analyses of all-cause mortality
Source: BMC Med. 2022 Aug 30;20:274. doi: 10.1186/s12916-022-02474-2 (PMC9425946; doi:10.1186/s12916-022-02474-2)
Supplement: Supplementary file 1 — Additional file 1: Full criteria used to identify individuals with mental disorders. Table S1. Frailty phenotype criteria. Table S2. Frailty index variables. Figure S1. Overlap between frailty phenotype and frailty index categories. Table S3. Sample characteristics stratified by sex. Figure S2. Frailty group differences stratified by sex. Table S4. Sex differences in frailty index. Figure S3. Sex-stratified regression models. Table S5. Sex-stratified regression models. Figure S4. Frailty phenotype by age. Table S6. Descriptive statistics follow-up. Figure S5. All-cause mortality by case status. Table S7. All-cause mortality by case status. Figure S6. All-cause mortality by frailty level. Table S8. All-cause mortality by frailty level. Figure S7. Survival probabilities by frailty phenotype and case status. Figure S8. Survival probabilities by frailty index categories and case status. Figure S9. All-cause mortality by frailty phenotype and case status. Figure S10. All-cause mortality by frailty index categories and case status. Figure S11. All-cause mortality by frailty phenotype and case status stratified by sex. Table S9. All-cause mortality by frailty phenotype and case status stratified by sex. Figure S12. All-cause mortality by frailty index categories and case status stratified by sex. Table S10. All-cause mortality by frailty index categories and case status stratified by sex. Table S11. Descriptive statistics follow-up, comorbidity depression and anxiety disorders. Table S12. All-cause mortality by frailty phenotype, comorbidity depression and anxiety disorders. Table S13. All-cause mortality by frailty index categories, comorbidity depression and anxiety disorders. [file 12916_2022_2474_MOESM1_ESM.pdf]

Additional file 1

This material accompanies the article

Frailty in individuals with depression, bipolar disorder and anxiety disorders: longitudinal analyses of all-cause mortality

Authors: Julian Mutz, Umamah Choudhury, Jinlong Zhao and Alexandru Dregan

Published in BMC Medicine.

Table of contents:

Full criteria used to identify individuals with mental disorders 2
Frailty phenotype criteria 4
Frailty index variables 5
Overlap between frailty phenotype and frailty index categories 7
Sample characteristics stratified by sex 8
Frailty group differences stratified by sex 10
Sex differences in frailty index 11
Sex-stratified regression models 12
Frailty phenotype by age 13
Descriptive statistics follow-up 14
All-cause mortality by case status 15
All-cause mortality by frailty level 16
Survival probabilities by frailty phenotype and case status 17
Survival probabilities by frailty index categories and case status 18
All-cause mortality by frailty phenotype and case status 19
All-cause mortality by frailty index categories and case status 20
All-cause mortality by frailty phenotype and case status stratified by sex 21
All-cause mortality by frailty index categories and case status stratified by sex 23
Sensitivity analysis 25
  Individuals with or without comorbid depression and anxiety disorders 25

Full criteria used to identify individuals with mental disorders

Criteria below are adapted from Mutz and Lewis (1) Mutz, Young and Lewis (2) and Mutz, Hoppen, Fabbri and Lewis (3).

Depression
Lifetime depression was assessed as part of the mental health follow-up questionnaire (MHQ) using a modified version of the depression module of the Composite International Diagnostic Interview Short Form (CIDI-SF) and defined according to DSM-5 criteria for major depressive disorder (4). To achieve maximum coverage of the UK Biobank study, we also included in our definition of lifetime depression individuals who had reported during the nurse-led interview at baseline that a doctor had told them that they had depression (UK Biobank data field 20002), participants who reported in response to a single-item question on the MHQ that a professional (doctors, nurse or person with specialist training such as a psychologist or a therapist) had diagnosed them with depression (field 20544), participants with a hospital inpatient record containing an ICD-10 code for depression (F32-F33), participants with a primary care record containing a Read v2 or CTV3 code for depression (see Fabbri et al. (2021) (5) for diagnostic codes and data extraction procedures), and those who were classified as individuals with probable depression according to Smith et al. (2013) (6) based on additional questions that were introduced during the later stages of the baseline assessment.

Bipolar disorder
Bipolar disorder was assessed with the MHQ that included several questions on mania and hypomania in combination with the CIDI-SF to assess lifetime depression (4). We also included in our definition of bipolar disorder individuals who had reported during the nurse-led interview at baseline that a doctor had told them that they had "mania/bipolar disorder/manic depression" (field 20002), participants who responded to a single-item question on the MHQ that a professional had diagnosed them with "mania, hypomania, bipolar or manic-depression" (field 20544), participants with a hospital inpatient record containing an ICD-10 code for mania or bipolar disorder (F30-F31), participants with a primary care record containing a Read v2 or CTV3 code for bipolar disorder (see Fabbri et al. (2021) (5)), and participants who were classified as individuals with probable bipolar disorder according to Smith et al. (2013) (6) based on additional questions that were introduced during the later stages of the baseline assessment.

Anxiety disorders
We used a transdiagnostic phenotype for lifetime anxiety disorders and identified cases from multiple sources: the MHQ which included the anxiety disorder module of the CIDI-SF and assessed generalised anxiety disorder according to DSM-5 criteria (4); individuals with a Generalised Anxiety Disorder Assessment (GAD-7) sum score of ≥10 (7), which was assessed as part of the MHQ; individuals who had reported "anxiety/panic attacks" during the nurse-led interview at baseline (field 20002), or "anxiety, nerves or generalized anxiety disorder", "social anxiety or social phobia", "any other phobia", "panic attacks" or "agoraphobia" in response to a single-item question on the MHQ (field 20544); participants with a hospital inpatient record containing an ICD-10 code for anxiety disorders (F40-F41); participants with at least two primary care records containing a Read v2 or CTV3 code for anxiety disorders (see Fabbri et al. (2021) (5)).

References

1. Mutz J, Lewis CM. Lifetime depression and age-related changes in body composition, cardiovascular function, grip strength and lung function: sex-specific analyses in the UK Biobank. Aging. 2021;13(13):17038-79.
2. Mutz J, Young AH, Lewis CM. Age-related changes in physiology in individuals with bipolar disorder. Journal of Affective Disorders. 2022;296:157-68.
3. Mutz J, Hoppen TH, Fabbri C, Lewis CM. Anxiety disorders and age-related changes in physiology. The British Journal of Psychiatry. 2022:1-10.

4. Davis KA, Coleman JR, Adams M, Allen N, Breen G, Cullen B, et al. Mental health in UK Biobank–development, implementation and results from an online questionnaire completed by 157 366 participants: a reanalysis. BJPsych Open. 2020;6(2):E18.
5. Fabbri C, Hagenaars SP, John C, Williams AT, Shrine N, Moles L, et al. Genetic and clinical characteristics of treatment-resistant depression using primary care records in two UK cohorts. Molecular Psychiatry. 2021.
6. Smith DJ, Nicholl BI, Cullen B, Martin D, Ul-Haq Z, Evans J, et al. Prevalence and characteristics of probable major depression and bipolar disorder within UK biobank: cross-sectional study of 172,751 participants. PloS One. 2013;8(11):e75362.
7. Plummer F, Manea L, Trepel D, McMillan D. Screening for anxiety disorders with the GAD-7 and GAD-2: a systematic review and diagnostic metaanalysis. General Hospital Psychiatry. 2016;39:24-31.

Frailty phenotype criteria

Table S1. Frailty phenotype criteria

Variable	UK Biobank question	UK Biobank data field	Coded in frailty phenotype[1]
Weight loss	"Compared with one year ago, has your weight changed?"	2306	Lost weight = 1<br>Other = 0
Exhaustion	"Over the past two weeks, how often have you felt tired or had little energy?"	120107	More than half the days = 1<br>Nearly every day = 1<br>Other = 0
Physical activity	"In the last 4 weeks did you spend any time doing the following [activities]?"	6164[2]<br>1011 (frequency light DIY)	No physical activity = 1<br>Light DIY (≤ 1/week) = 1<br>Light DIY (≥ 2-3/week) = 0<br>Heavy DIY = 0<br>Walking for pleasure = 0<br>Other exercises = 0<br>Strenuous sports = 0
Walking speed	"How would you describe your usual walking pace?"	924	Slow = 1<br>Other = 0
Hand-grip strength	Measured during the baseline assessment	46 (left hand)<br>47 (right hand)<br>1707 (handedness)<br>31 (sex)<br>21001 (BMI)	Maximal grip strength of self-reported dominant hand (adjusted for sex and BMI)[3].<br>Below 20th %tile = 1<br>Equal or above 20th %tile = 0

Note: BMI = body mass index; DIY = do-it-yourself. [1] "do not know" or "prefer not to answer" were coded as missing data. [2] array variables. [3] grip strength was regressed on sex and BMI, and the residuals from this regression were used to identify individuals in the bottom 20% of the distribution. Criteria adapted from frailty phenotype by Fried et al. (2001), doi: 10.1093/gerona/56.3.m146 and Hanlon et al. (2018), doi: 10.1016/S2468-2667(18)30091-4.

Frailty index variables

Table S2. Variables included in the frailty index

Type of deficit	Item	Variable	UK Biobank data field[1]	Coded in frailty index
Sensory	1	Glaucoma[2]	6148[3]<br>2227<br>20002[3] (1277)	Yes = 1<br>No = 0
	2	Cataracts[2]	6148[3]<br>2227<br>20002[3] (1278)	Yes = 1<br>No = 0
	3	Hearing difficulty/problems	2247	Yes = 1<br>I am completely deaf = 1<br>No = 0
Cranial	4	Migraine[2]	2473<br>20002[3] (1265)	Yes = 1<br>No = 0
	5	Mouth/teeth dental problems	6149	Any = 1<br>None of the above = 0
Mental wellbeing	6	Overall health rating	2178	Poor = 1<br>Fair = 0.5<br>Good = 0.25<br>Excellent = 0
	7	Frequency of tiredness/lethargy in last two weeks	2080	Nearly every day = 1<br>More than half the days = 0.5<br>Several days = 0.25<br>None = 0
	8	Sleeplessness/insomnia	1200	Usually = 1<br>Sometimes = 0.5<br>Never/rarely = 0
	9	Frequency of depressed mood in last two weeks	2050	Nearly every day = 1<br>More than half the days = 0.75<br>Several days = 0.5<br>Not at all = 0
	10	Nervous feelings	1970	Yes = 1<br>No = 0
	11	Anxiety / panic attacks[2]	2473<br>20002[3] (1287)	Yes = 1<br>No = 0
	12	Loneliness, isolation	2020	Yes = 1<br>No = 0
	13	Miserableness	1930	Yes = 1<br>No = 0
Infirmity	14	Long-standing illness, disability or infirmity	2188	Yes = 1<br>No = 0
	15	Falls in the last year	2296	More than one fall = 1<br>Only one fall = 0.5<br>No falls = 0
	16	Fractured/broken bones in last five years	2463	Yes = 1<br>No = 0
Cardiometabolic	17	Diabetes[2]	2443<br>20002[3] (1220 & 1223)	Yes = 1<br>No = 0
	18	Myocardial infarction[2]	6150[3]<br>20002[3] (1075)	Yes = 1<br>No = 0
	19	Angina[2]	6150[3]<br>20002[3] (1074)	Yes = 1<br>No = 0
	20	Stroke / ischaemic stroke[2]	6150[3]<br>20002[3] (1081 & 1583)	Yes = 1<br>No = 0
	21	Hypertension[2]	6150[3]<br>20002[3] (1065)	Yes = 1<br>No = 0
	22	Hypothyroidism[2]	2473<br>20002[3] (1226)	Yes = 1<br>No = 0
	23	Deep venous thrombosis[2]	6152[3]<br>20002[3] (1094)	Yes = 1<br>No = 0
	24	Cholesterol lowering medication use	6153 (Females)<br>6177 (Males)	Yes = 1<br>No = 0
Respiratory	25	Wheeze or whistling in the chest in last year	2316	Yes = 1<br>No = 0
	26	Pneumonia[2]	2473<br>20002[3] (1398)	Yes = 1<br>No = 0
	27	Emphysema / chronic bronchitis[2]	6152[3]<br>20002[3] (1113)	Yes = 1<br>No = 0
	28	Asthma[2]	6152[3]	Yes = 1

			20002[3] (1111)	No = 0
Musculoskeletal	29	Rheumatoid arthritis[2]	2473<br>20002[3] (1464)	Yes = 1<br>No = 0
	30	Osteoarthritis[2]	2473<br>20002[3] (1465)	Yes = 1<br>No = 0
	31	Gout[2]	2473<br>20002[3] (1466)	Yes = 1<br>No = 0
	32	Osteoporosis[2]	2473<br>20002[3] (1309)	Yes = 1<br>No = 0
Immunological	33	Hay fever / allergic rhinitis or eczema/dermatitis[2]	6152[3]<br>20002[3] (1387 & 1452)	Yes = 1<br>No = 0
	34	Psoriasis[2]	2473<br>20002[3] (1453)	Yes = 1<br>No = 0
Cancer	35	Any cancer diagnosis[2]	134<br>2453	At least 1 = 1<br>Yes = 1<br>No = 0
	36	Multiple cancers diagnosed[2]	134	More than 1 = 1<br>0 or 1 = 0
Pain	37	Chest pain or discomfort	2335	Yes = 1<br>No = 0
	38	Headache / neck or shoulder pain (in last month)	6159[3]	Yes = 1<br>No = 0
	39	Back pain (in last month)	6159[3]	Yes = 1<br>No = 0
	40	Stomach or abdominal pain (in last month)	6159[3]	Yes = 1<br>No = 0
	41	Hip pain (in last month)	6159[3]	Yes = 1<br>No = 0
	42	Knee pain (in last month)	6159[3]	Yes = 1<br>No = 0
	43	Pain all over the body (in last month)	6159[3]	Yes = 1<br>No = 0
	44	Facial pain (in last month)	6159[3]	Yes = 1<br>No = 0
	45	Sciatica[2]	2473<br>20002[3] (1476)	Yes = 1<br>No = 0
Gastrointestinal	46	Gastro-oesophageal reflux / gastric reflux[2]	2473<br>20002[3] (1138)	Yes = 1<br>No = 0
	47	Hiatus hernia[2]	2473<br>20002[3] (1474)	Yes = 1<br>No = 0
	48	Cholelithiasis / gall stones[2]	2473<br>20002[3] (1162)	Yes = 1<br>No = 0
	49	Diverticular disease / diverticulitis[2]	2473<br>20002[3] (1458)	Yes = 1<br>No = 0

Note: Variables included in frailty index from Williams et al. (2019), doi: 10.1093/gerona/gly094. [1] numbers shown in parentheses are UK Biobank diagnostic codes. [2] conditions diagnosed by a doctor. [3] array variables. For all items with only one corresponding data field, including item 24, “do not know” or “prefer not to answer” responses were coded as missing data. For items with multiple corresponding data fields, we first coded “Yes” and “No” responses using the self-report items and, including for data field 2473, “prefer not to answer” responses as missing data. Additional “Yes” responses were then ascertained from the nurse-led interview diagnostic codes (data field 20002). Individuals with missing data for both data fields 2473 and 20002 were coded as missing data. Items 1 and 2 were coded as “No” if data field 2227 was coded “No” or if data field 6148 was coded “do not know” and data field 20002 was coded “No”. Item 17 was coded as “No” if data field 2443 was coded “do not know” and data field 20002 was coded “No”. For item 35, “do not know” or “prefer not to answer” responses from data field 2453 were coded as missing data unless participants had at least one cancer for data field 134. For data fields, only data from the first instance (i.e., the baseline assessment) were used.

Overlap between frailty phenotype and frailty index categories

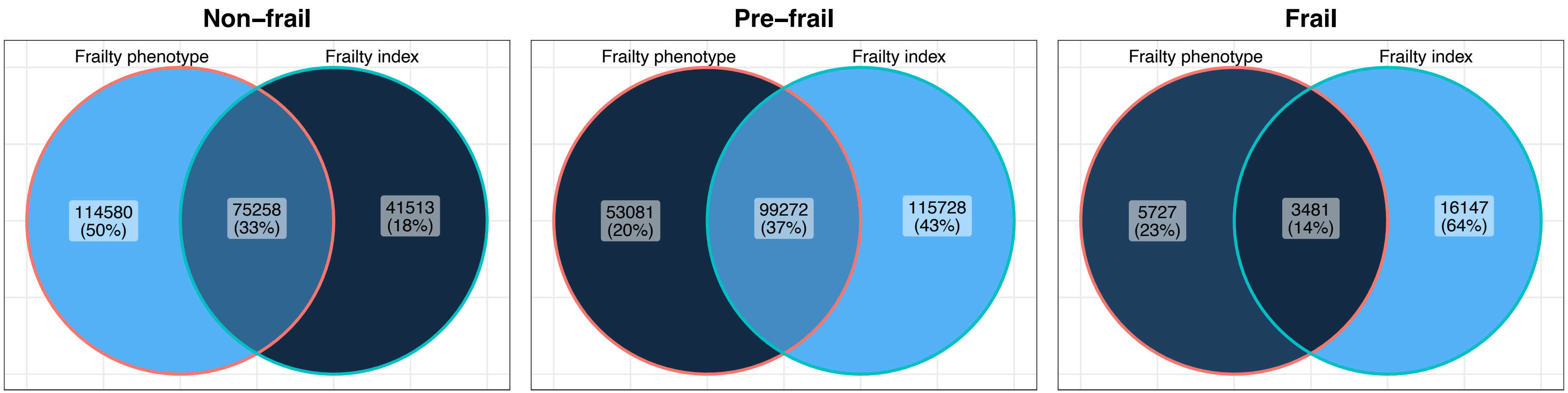

Figure S1. Venn diagram showing the overlap between individuals with different frailty levels according to the frailty phenotype and frailty index categories. Cut-offs for frailty index categories were non frail (≤ 0.08), pre-frail (> 0.08 and < 0.25) and frail (≥ 0.25).

Sample characteristics stratified by sex

Table S3. Sample characteristics of individuals with and without mental disorders stratified by sex

	**Depression**		**Bipolar disorder**		**Anxiety disorder**		**No disorder**	
	Males	Females	Males	Females	Males	Females	Males	Females
	N=26546	N=50040	N=1319	N=1710	N=12835	N=24944	N=111347	N=109447
**Age**								
Mean (SD)	56.01 (8.04)	54.67 (7.81)	55.38 (8.28)	53.49 (7.68)	56.43 (7.95)	55.35 (7.84)	56.67 (8.27)	56.19 (8.01)
**Neighbourhood deprivation**								
Mean (SD)	-1.48 (2.92)	-1.48 (2.90)	-1.21 (2.99)	-1.01 (3.04)	-1.64 (2.88)	-1.61 (2.86)	-1.78 (2.83)	-1.77 (2.82)
**Ethnicity**								
White	25654 (96.6%)	48214 (96.4%)	1247 (94.5%)	1608 (94.0%)	12481 (97.2%)	24273 (97.3%)	105743 (95.0%)	103508 (94.6%)
Mixed-race	129 (0.5%)	393 (0.8%)	14 (1.1%)	14 (0.8%)	52 (0.4%)	139 (0.6%)	459 (0.4%)	646 (0.6%)
Black	148 (0.6%)	465 (0.9%)	10 (0.8%)	31 (1.8%)	51 (0.4%)	197 (0.8%)	1479 (1.3%)	1801 (1.6%)
Asian	415 (1.6%)	540 (1.1%)	30 (2.3%)	37 (2.2%)	176 (1.4%)	175 (0.7%)	2513 (2.3%)	2008 (1.8%)
Chinese	31 (0.1%)	89 (0.2%)	4 (0.3%)	2 (0.1%)	15 (0.1%)	27 (0.1%)	322 (0.3%)	500 (0.5%)
Other	169 (0.6%)	339 (0.7%)	14 (1.1%)	18 (1.1%)	60 (0.5%)	133 (0.5%)	831 (0.7%)	984 (0.9%)
**Highest qualification**								
None	3836 (14.5%)	6806 (13.6%)	147 (11.1%)	169 (9.9%)	1737 (13.5%)	3460 (13.9%)	17361 (15.6%)	18308 (16.7%)
O levels/GCSEs/CSEs	6495 (24.5%)	14814 (29.6%)	316 (24.0%)	478 (28.0%)	3008 (23.4%)	7457 (29.9%)	27863 (25.0%)	33649 (30.7%)
A levels/NVQ/HND/HNC[1]	6346 (23.9%)	11759 (23.5%)	308 (23.4%)	406 (23.7%)	3087 (24.1%)	5844 (23.4%)	27070 (24.3%)	24198 (22.1%)
Degree	9869 (37.2%)	16661 (33.3%)	548 (41.5%)	657 (38.4%)	5003 (39.0%)	8183 (32.8%)	39053 (35.1%)	33292 (30.4%)
**Spouse/partner cohabitation**								
No	2418 (9.1%)	8446 (16.9%)	149 (11.3%)	372 (21.8%)	1041 (8.1%)	3695 (14.8%)	6839 (6.1%)	12309 (11.2%)
Yes	24128 (90.9%)	41594 (83.1%)	1170 (88.7%)	1338 (78.2%)	11794 (91.9%)	21249 (85.2%)	104508 (93.9%)	97138 (88.8%)
**Smoking status**								
Never	11985 (45.1%)	27637 (55.2%)	549 (41.6%)	859 (50.2%)	5884 (45.8%)	13984 (56.1%)	57470 (51.6%)	69387 (63.4%)
Former	11075 (41.7%)	17246 (34.5%)	534 (40.5%)	588 (34.4%)	5424 (42.3%)	8704 (34.9%)	42640 (38.3%)	32725 (29.9%)
Current	3486 (13.1%)	5157 (10.3%)	236 (17.9%)	263 (15.4%)	1527 (11.9%)	2256 (9.0%)	11237 (10.1%)	7335 (6.7%)
**Alcohol intake frequency**								
Never	1850 (7.0%)	4392 (8.8%)	140 (10.6%)	189 (11.1%)	882 (6.9%)	2281 (9.1%)	5313 (4.8%)	8998 (8.2%)
Special occasions	2069 (7.8%)	7418 (14.8%)	107 (8.1%)	303 (17.7%)	890 (6.9%)	3662 (14.7%)	6941 (6.2%)	14413 (13.2%)
1-3/month	2646 (10.0%)	6668 (13.3%)	117 (8.9%)	235 (13.7%)	1172 (9.1%)	3084 (12.4%)	9269 (8.3%)	13411 (12.3%)
1-2/week	6489 (24.4%)	12612 (25.2%)	295 (22.4%)	429 (25.1%)	3071 (23.9%)	6200 (24.9%)	29518 (26.5%)	29679 (27.1%)
3-4/week	6600 (24.9%)	10366 (20.7%)	314 (23.8%)	264 (15.4%)	3256 (25.4%)	5298 (21.2%)	31255 (28.1%)	24668 (22.5%)
Daily/almost daily	6892 (26.0%)	8584 (17.2%)	346 (26.2%)	290 (17.0%)	3564 (27.8%)	4419 (17.7%)	29051 (26.1%)	18278 (16.7%)
**Body mass index**								
Mean (SD)	28.14 (4.39)	27.44 (5.38)	28.51 (4.66)	27.88 (5.84)	27.85 (4.27)	27.10 (5.26)	27.67 (3.96)	26.69 (4.83)
**Systolic blood pressure**								
Mean (SD)	139.23 (16.74)	132.76 (18.47)	137.58 (16.81)	130.86 (17.65)	139.91 (16.84)	133.81 (18.73)	141.31 (17.35)	135.92 (19.43)
**Diastolic blood pressure**								
Mean (SD)	83.78 (9.93)	80.35 (9.92)	83.38 (10.08)	80.10 (9.97)	83.94 (9.84)	80.49 (9.90)	84.18 (9.88)	80.78 (9.95)
**Cholesterol**								
Mean (SD)	5.49 (1.14)	5.84 (1.12)	5.49 (1.14)	5.81 (1.14)	5.49 (1.12)	5.86 (1.12)	5.53 (1.11)	5.88 (1.12)
**Multimorbidity count**								

None	4160 (15.7%)	8529 (17.0%)	171 (13.0%)	209 (12.2%)	1960 (15.3%)	3970 (15.9%)	31250 (28.1%)	31266 (28.6%)
One	6301 (23.7%)	11948 (23.9%)	275 (20.8%)	376 (22.0%)	3002 (23.4%)	5788 (23.2%)	31513 (28.3%)	31147 (28.5%)
Two	5474 (20.6%)	10286 (20.6%)	272 (20.6%)	348 (20.4%)	2721 (21.2%)	5218 (20.9%)	22275 (20.0%)	21283 (19.4%)
Three	4059 (15.3%)	7597 (15.2%)	242 (18.3%)	274 (16.0%)	2025 (15.8%)	3909 (15.7%)	13069 (11.7%)	12402 (11.3%)
Four	2765 (10.4%)	4720 (9.4%)	136 (10.3%)	206 (12.0%)	1342 (10.5%)	2442 (9.8%)	6800 (6.1%)	6574 (6.0%)
Five or more	3787 (14.3%)	6960 (13.9%)	223 (16.9%)	297 (17.4%)	1785 (13.9%)	3617 (14.5%)	6440 (5.8%)	6775 (6.2%)
**Antidepressant use**								
No	21687 (81.7%)	38639 (77.2%)	988 (74.9%)	1187 (69.4%)	10598 (82.6%)	19271 (77.3%)	111347 (100.0%)	109447 (100.0%)
Yes	4859 (18.3%)	11401 (22.8%)	331 (25.1%)	523 (30.6%)	2237 (17.4%)	5673 (22.7%)	0 (0.0%)	0 (0.0%)
**Antipsychotic use**								
No	26433 (99.6%)	49843 (99.6%)	1245 (94.4%)	1582 (92.5%)	12764 (99.4%)	24822 (99.5%)	111347 (100.0%)	109447 (100.0%)
Yes	113 (0.4%)	197 (0.4%)	74 (5.6%)	128 (7.5%)	71 (0.6%)	122 (0.5%)	0 (0.0%)	0 (0.0%)
**Lithium use**								
No	26514 (99.9%)	49972 (99.9%)	1184 (89.8%)	1571 (91.9%)	12824 (99.9%)	24916 (99.9%)	111347 (100.0%)	109447 (100.0%)
Yes	32 (0.1%)	68 (0.1%)	135 (10.2%)	139 (8.1%)	11 (0.1%)	28 (0.1%)	0 (0.0%)	0 (0.0%)
**Frailty phenotype**								
Non-frail	12843 (48.4%)	22630 (45.2%)	591 (44.8%)	693 (40.5%)	6402 (49.9%)	11605 (46.5%)	65107 (58.5%)	60873 (55.6%)
Pre-frail	12554 (47.3%)	24995 (50.0%)	668 (50.6%)	911 (53.3%)	5971 (46.5%)	12228 (49.0%)	44613 (40.1%)	46268 (42.3%)
Frail	1149 (4.3%)	2415 (4.8%)	60 (4.5%)	106 (6.2%)	462 (3.6%)	1111 (4.5%)	1627 (1.5%)	2306 (2.1%)
**Frailty phenotype count**								
None	12843 (48.4%)	22630 (45.2%)	591 (44.8%)	693 (40.5%)	6402 (49.9%)	11605 (46.5%)	65107 (58.5%)	60873 (55.6%)
One	9294 (35.0%)	18266 (36.5%)	493 (37.4%)	650 (38.0%)	4544 (35.4%)	9034 (36.2%)	36308 (32.6%)	36371 (33.2%)
Two	3260 (12.3%)	6729 (13.4%)	175 (13.3%)	261 (15.3%)	1427 (11.1%)	3194 (12.8%)	8305 (7.5%)	9897 (9.0%)
Three	926 (3.5%)	1944 (3.9%)	46 (3.5%)	88 (5.1%)	372 (2.9%)	892 (3.6%)	1398 (1.3%)	1978 (1.8%)
Four	206 (0.8%)	426 (0.9%)	12 (0.9%)	18 (1.1%)	82 (0.6%)	195 (0.8%)	216 (0.2%)	307 (0.3%)
Five	17 (0.1%)	45 (0.1%)	2 (0.2%)	0 (0.0%)	8 (0.1%)	24 (0.1%)	13 (0.0%)	21 (0.0%)
Mean (SD)	0.73 (0.87)	0.79 (0.89)	0.79 (0.88)	0.88 (0.92)	0.69 (0.84)	0.76 (0.87)	0.52 (0.71)	0.58 (0.76)
**Frailty index**								
Mean (SD)	0.15 (0.08)	0.15 (0.08)	0.16 (0.08)	0.17 (0.08)	0.15 (0.08)	0.15 (0.08)	0.10 (0.06)	0.11 (0.06)
**Frailty index categories**								
Non-frail	5168 (19.5%)	9229 (18.4%)	208 (15.8%)	237 (13.9%)	2401 (18.7%)	4240 (17.0%)	46028 (41.3%)	42161 (38.5%)
Pre-frail	18213 (68.6%)	35067 (70.1%)	908 (68.8%)	1190 (69.6%)	8946 (69.7%)	17635 (70.7%)	62127 (55.8%)	63521 (58.0%)
Frail	3165 (11.9%)	5744 (11.5%)	203 (15.4%)	283 (16.5%)	1488 (11.6%)	3069 (12.3%)	3192 (2.9%)	3765 (3.4%)

Note: SD = standard deviation; GCSEs = general certificate of secondary education; CSE = certificate of secondary education; NVQ = national vocational qualification; HND = higher national diploma; HNC = higher national certificate. [1] also includes 'other professional qualifications'. Cut-offs for frailty index categories were non frail (≤ 0.08), pre-frail (> 0.08 and < 0.25) and frail (≥ 0.25).

Frailty group differences stratified by sex

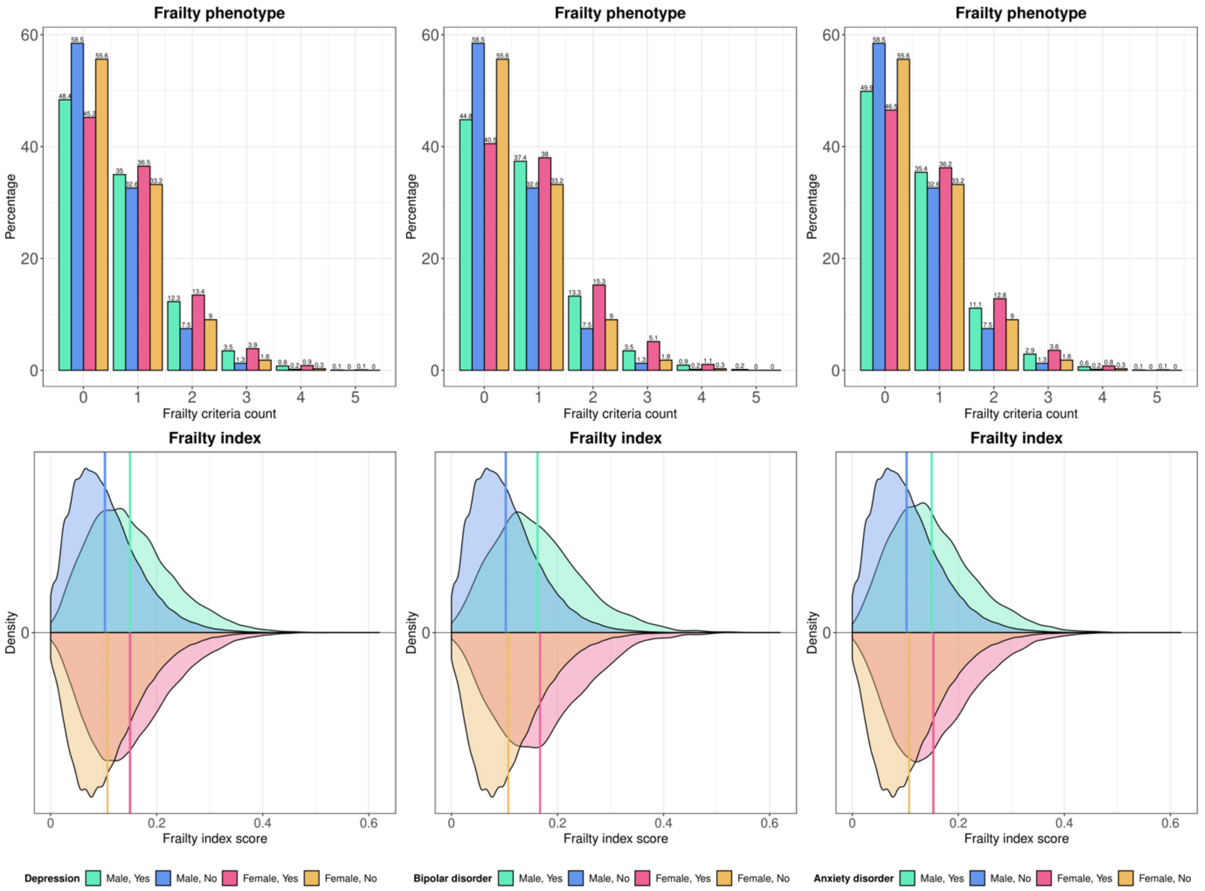

Figure S2. Histograms and density plots showing the distribution of the frailty phenotype criteria count (top panels) and the frailty index (bottom panels), respectively, for individuals with and without mental disorders stratified by sex.

Sex differences in frailty index

Table S4. Sex differences in the frailty index

Term	SMD	95% CI		$p_{Bonf.}$	p_{BH}
No mental disorder	0.072	0.064	0.081	<0.001	<0.001
Depression	0.001	-0.014	0.016	>0.999	0.891
Bipolar disorder	0.055	-0.017	0.127	0.536	0.179
Anxiety disorder	0.039	0.017	0.060	0.002	<0.001

Note: SMD = standardised mean difference; CI = confidence interval; Bonf. = Bonferroni; BH = Benjamini & Hochberg.

Sex-stratified regression models

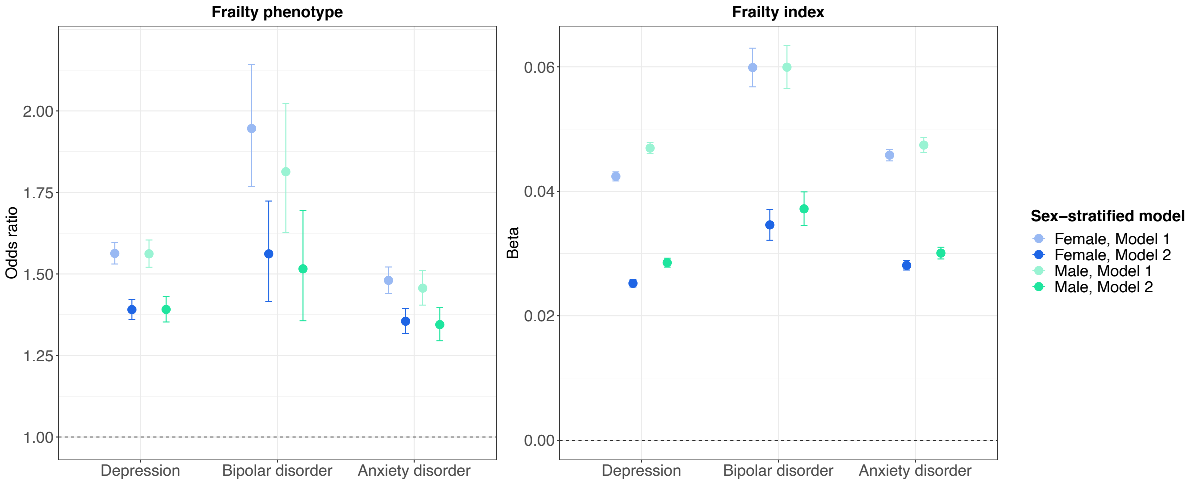

Figure S3. Frailty in individuals with mental disorders compared to individuals without mental disorders (reference group) stratified by sex. Estimates shown for the frailty phenotype are odds ratios and 95% confidence intervals (CI) from ordinal logistic regression models, indicating changes in odds of being frailer associated with being in the case group relative to the comparison group without mental disorders. Estimates shown for the frailty index are ordinary least squares regression beta coefficients and 95% CI. Model 1 – unadjusted; Model 2 – adjusted for age, sex, ethnicity, highest qualification, Townsend deprivation index, cohabitation with spouse/partner, smoking status, alcohol intake frequency, systolic and diastolic blood pressure, body mass index, cholesterol, multimorbidity count and assessment centre.

Table S5. Frailty in males and females with and without mental disorders

	**Males**						**Females**					
	Model 1			Model 2			Model 1			Model 2		
**Frailty phenotype**	OR	95% CI		OR	95% CI		OR	95% CI		OR	95% CI	
No mental disorder	Ref	-	-	Ref	-	-	Ref	-	-	Ref	-	-
Depression	1.562	1.521	1.604	1.391	1.353	1.431	1.563	1.531	1.596	1.391	1.360	1.422
Bipolar disorder	1.814	1.627	2.022	1.516	1.356	1.694	1.946	1.768	2.143	1.562	1.415	1.724
Anxiety disorder	1.457	1.404	1.511	1.345	1.295	1.396	1.480	1.441	1.521	1.355	1.317	1.394
**Frailty index**	β	95% CI		β	95% CI		β	95% CI		β	95% CI	
No mental disorder	Ref	-	-	Ref	-	-	Ref	-	-	Ref	-	-
Depression	0.047	0.046	0.048	0.029	0.028	0.029	0.042	0.042	0.043	0.025	0.025	0.026
Bipolar disorder	0.060	0.056	0.063	0.037	0.034	0.040	0.060	0.057	0.063	0.035	0.032	0.037
Anxiety disorder	0.047	0.046	0.049	0.030	0.029	0.031	0.046	0.045	0.047	0.028	0.027	0.029

Note: OR = odds ratio; β = ordinary least squares regression beta coefficient; CI = confidence interval; Ref = reference group. All Bonferroni-adjusted p-values < 0.001. Odds ratios indicate changes in odds of being frailer associated with being in the case group relative to the comparison group without mental disorders. Model 1 – unadjusted; Model 2 – adjusted for age, sex, ethnicity, highest qualification, Townsend deprivation index, cohabitation with spouse/partner, smoking status, alcohol intake frequency, systolic and diastolic blood pressure, body mass index, cholesterol, multimorbidity count and assessment centre.

Frailty phenotype by age

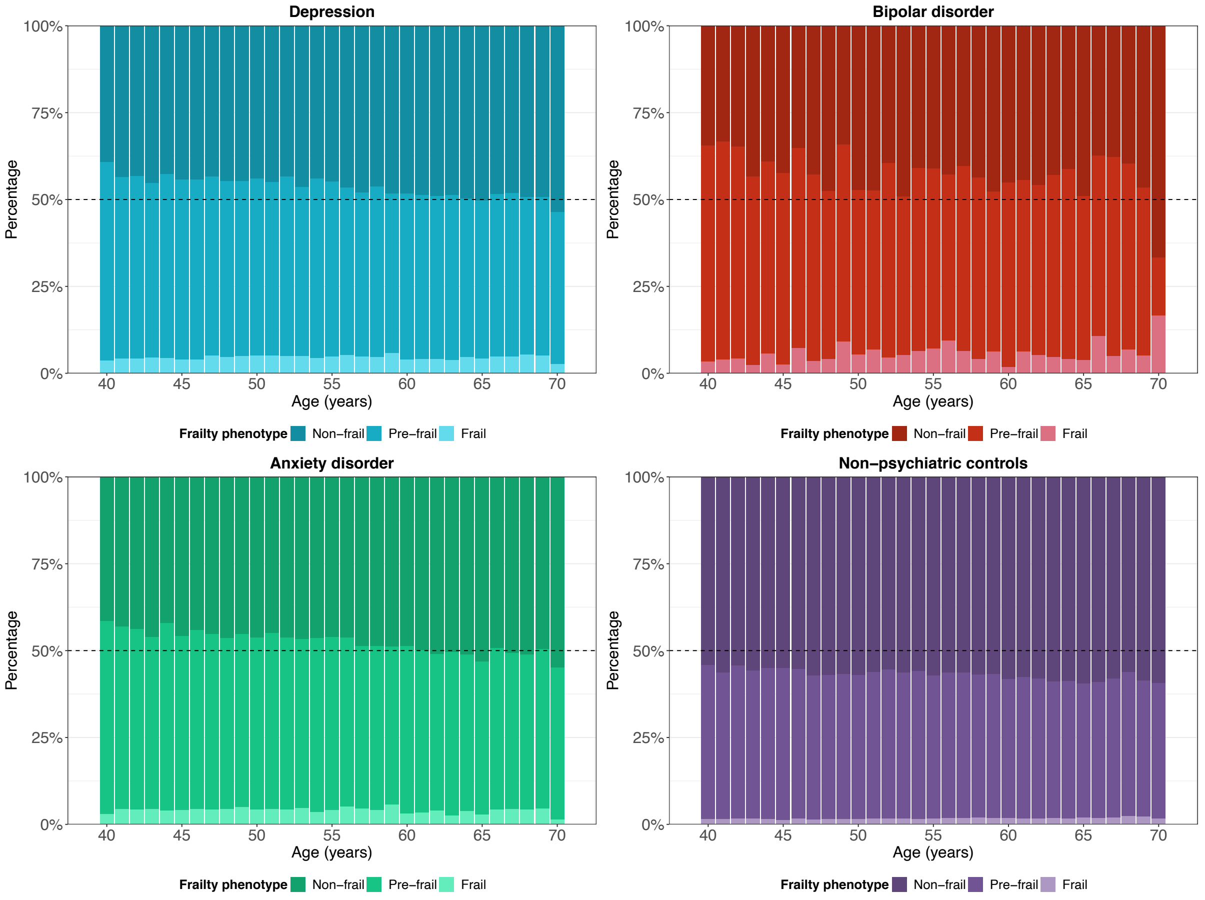

Figure S4. Stacked bar charts showing the frailty phenotype by age in individuals with and without mental disorders.

Descriptive statistics follow-up

Table S6. Descriptive statistics follow-up

		Depression		Bipolar disorder		Anxiety disorder	
Full sample size (cases)		297380 (76586)		223823 (3029)		258573 (37779)	
Person-years follow-up		3516706		2654566		3066828	
Median (IQR) censored		12.09 (1.35)		12.19 (1.31)		12.14 (1.31)	
Deaths, all (cases)		17240 (4138)		13315 (213)		15052 (1950)	
**Frailty phenotype**		Censored	Deaths	Censored	Deaths	Censored	Deaths
Non-frail	No	119473	6507	119473	6507	119473	6507
Non-frail	Yes	33898	1575	1209	75	17184	823
Pre-frail	No	84865	6016	84865	6016	84865	6016
Pre-frail	Yes	35438	2111	1463	116	17264	935
Frail	No	3354	579	3354	579	3354	579
Frail	Yes	3112	452	144	22	1381	192
**Frailty index categories**							
Non-frail	No	84753	3436	84753	3436	84753	3436
Non-frail	Yes	13927	470	428	17	6408	233
Pre-frail	No	117045	8603	117045	8603	117045	8603
Pre-frail	Yes	50584	2696	1960	138	25289	1292
Frail	No	5894	1063	5894	1063	5894	1063
Frail	Yes	7937	972	428	58	4132	425

Note: IQR = interquartile range.

All-cause mortality by case status

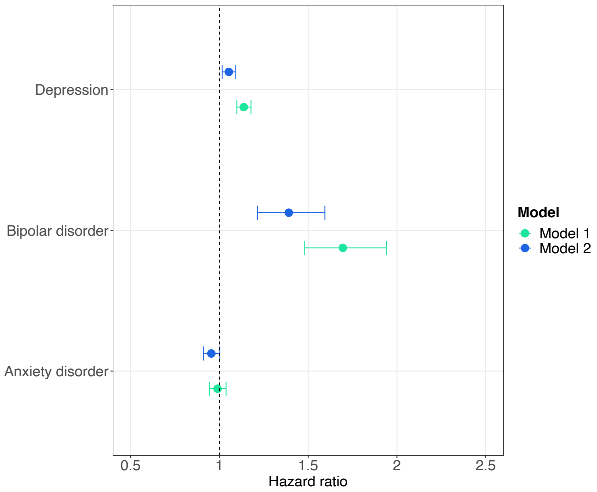

Figure S5. All-cause mortality in individuals with mental disorders and without mental disorders (reference group). Hazard ratios (HR) with 95% confidence intervals from Cox proportional hazards models. Age (in years) was used as the underlying time axis. Model 1 – unadjusted; Model 2 – adjusted for sex, ethnicity, highest qualification, Townsend deprivation index, cohabitation with spouse/partner, smoking status, alcohol intake frequency, systolic and diastolic blood pressure, body mass index, cholesterol, multimorbidity count and assessment centre.

Table S7. All-cause mortality by case status

	Model 1					Model 2				
	HR	95% CI		$p_{Bonf.}$	p_{BH}	HR	95% CI		$p_{Bonf.}$	p_{BH}
No mental disorder	Ref	-	-	-	-	Ref	-	-	-	-
Depression	1.137	1.098	1.178	<0.001	<0.001	1.053	1.015	1.092	0.372	0.074
Bipolar disorder	1.696	1.481	1.941	<0.001	<0.001	1.391	1.213	1.594	<0.001	<0.001
Anxiety disorder	0.989	0.943	1.038	>0.999	0.658	0.955	0.909	1.002	0.033	0.008

Note: HR = hazard ratio; CI = confidence interval; Ref = reference group; Bonf. = Bonferroni; BH = Benjamini & Hochberg. Age (in years) was used as the underlying time axis. Model 1 – unadjusted; Model 2 – adjusted for sex, ethnicity, highest qualification, Townsend deprivation index, cohabitation with spouse/partner, smoking status, alcohol intake frequency, systolic and diastolic blood pressure, body mass index, cholesterol, multimorbidity count and assessment centre.

All-cause mortality by frailty level

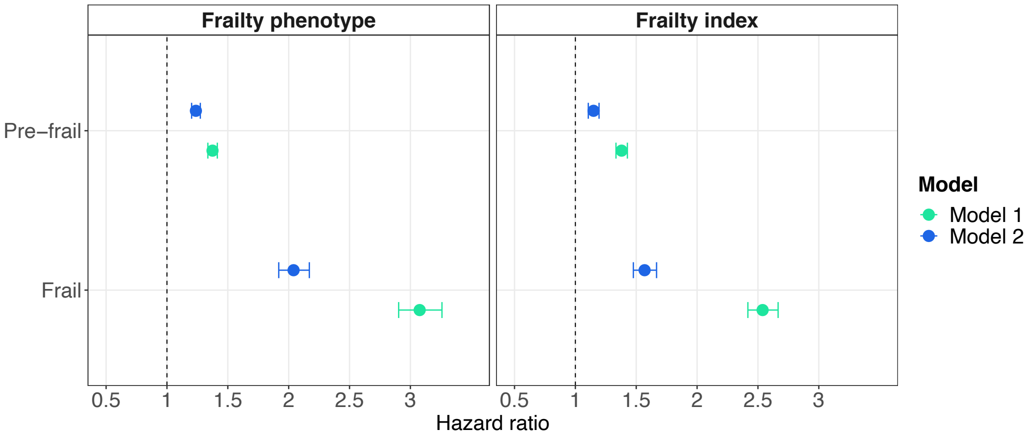

Figure S6. All-cause mortality by frailty level (non-frail, pre-frail and frail). Reference group: non-frail participants. Hazard ratios (HR) with 95% confidence intervals from Cox proportional hazards models. Age (in years) was used as the underlying time axis. Model 1 – unadjusted; Model 2 – adjusted for sex, ethnicity, highest qualification, Townsend deprivation index, cohabitation with spouse/partner, smoking status, alcohol intake frequency, systolic and diastolic blood pressure, body mass index, cholesterol, multimorbidity count and assessment centre.

Table S8. All-cause mortality by frailty level

	Model 1					Model 2				
**Frailty phenotype**	HR	95% CI		$p_{Bonf.}$	p_{BH}	HR	95% CI		$p_{Bonf.}$	p_{BH}
Non-frail	Ref	-	-	-	-	Ref	-	-	-	-
Pre-frail	1.374	1.336	1.414	<0.001	<0.001	1.238	1.202	1.274	<0.001	<0.001
Frail	3.076	2.904	3.259	<0.001	<0.001	2.040	1.918	2.170	<0.001	<0.001
**Frailty index categories**										
Non-frail	Ref	-	-	-	-	Ref	-	-	-	-
Pre-frail	1.380	1.334	1.427	<0.001	<0.001	1.150	1.106	1.194	<0.001	<0.001
Frail	2.538	2.417	2.666	<0.001	<0.001	1.569	1.477	1.667	<0.001	<0.001

Note: HR = hazard ratio; CI = confidence interval; Ref = reference group; Bonf. = Bonferroni; BH = Benjamini & Hochberg. Age (in years) was used as the underlying time axis. Model 1 – unadjusted; Model 2 – adjusted for sex, ethnicity, highest qualification, Townsend deprivation index, cohabitation with spouse/partner, smoking status, alcohol intake frequency, systolic and diastolic blood pressure, body mass index, cholesterol, multimorbidity count and assessment centre.

Survival probabilities by frailty phenotype and case status

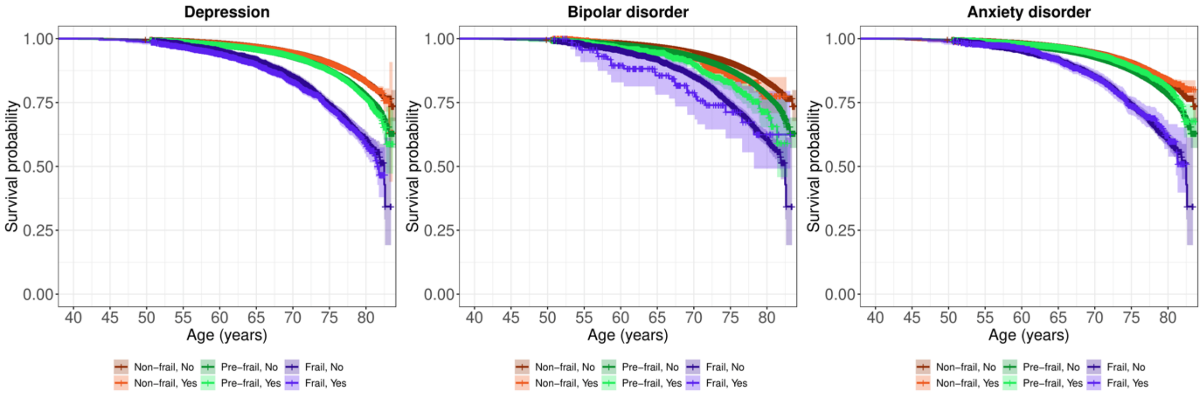

Figure S7. Kaplan-Meier survival probabilities for all-cause mortality by frailty phenotype (non-frail, pre-frail and frail) in individuals with mental disorders (yes) and without mental disorders (no). Two observations (one left panel and one right panel) were removed due to the death occurring after the maximum censoring age. All log-rank test p-values < 0.001.

Survival probabilities by frailty index categories and case status

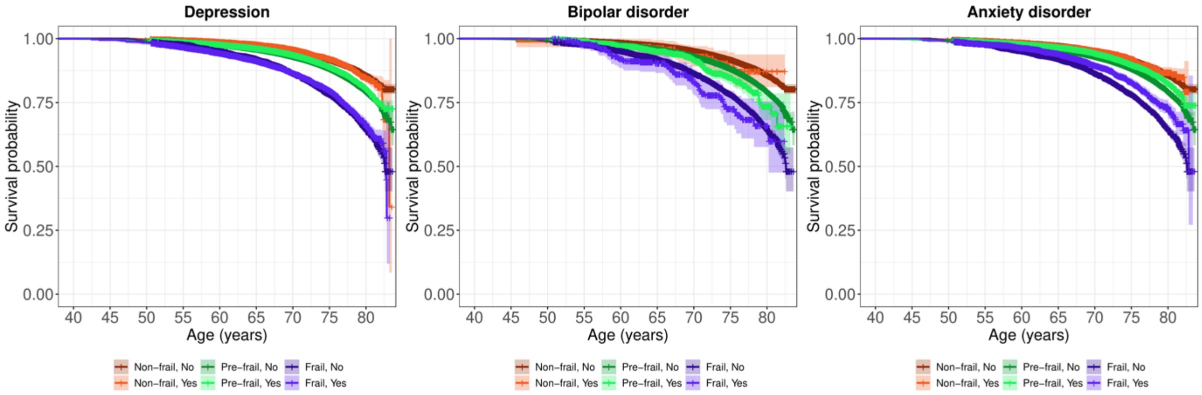

Figure S8. Kaplan-Meier survival probabilities for all-cause mortality by frailty index categories (non-frail, pre-frail and frail) in individuals with mental disorders (yes) and without mental disorders (no). All log-rank test *p*-values < 0.001.

All-cause mortality by frailty phenotype and case status

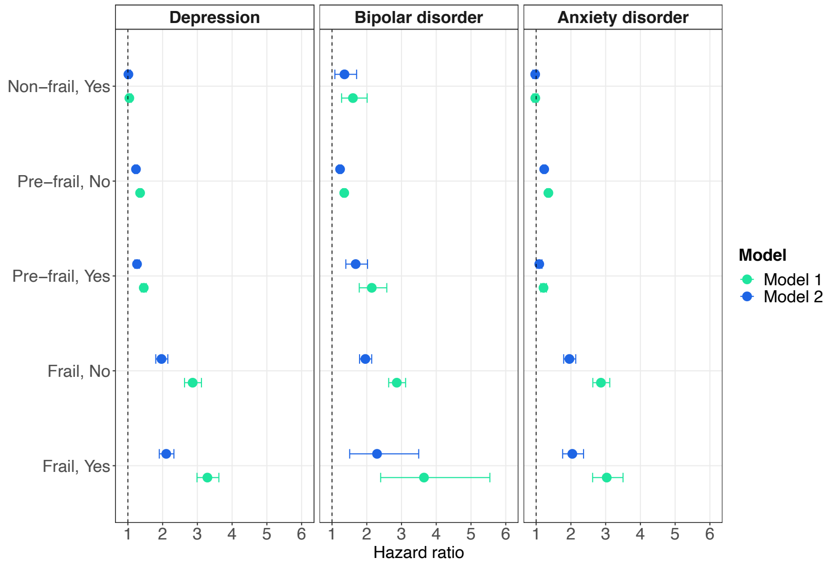

Figure S9. All-cause mortality by frailty phenotype (non-frail, pre-frail and frail) in individuals with and without mental disorders. Reference group: non-frail individuals without mental disorders. Hazard ratios (HR) with 95% confidence intervals from Cox proportional hazards models. Age (in years) was used as the underlying time axis. Model 1 – unadjusted; Model 2 – adjusted for sex, ethnicity, highest qualification, Townsend deprivation index, cohabitation with spouse/partner, smoking status, alcohol intake frequency, systolic and diastolic blood pressure, body mass index, cholesterol, multimorbidity count and assessment centre.

All-cause mortality by frailty index categories and case status

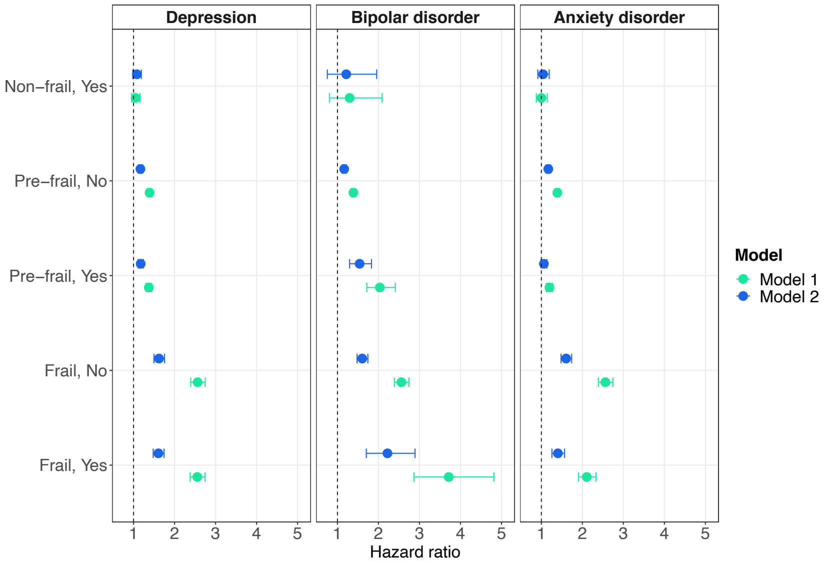

Figure S10. All-cause mortality by frailty index categories in individuals with and without mental disorders. Reference group: non-frail individuals without mental disorders. Hazard ratios (HR) with 95% confidence intervals from Cox proportional hazards models. Age (in years) was used as the underlying time axis. Model 1 – unadjusted; Model 2 – adjusted for sex, ethnicity, highest qualification, Townsend deprivation index, cohabitation with spouse/partner, smoking status, alcohol intake frequency, systolic and diastolic blood pressure, body mass index, cholesterol, multimorbidity count and assessment centre.

All-cause mortality by frailty phenotype and case status stratified by sex

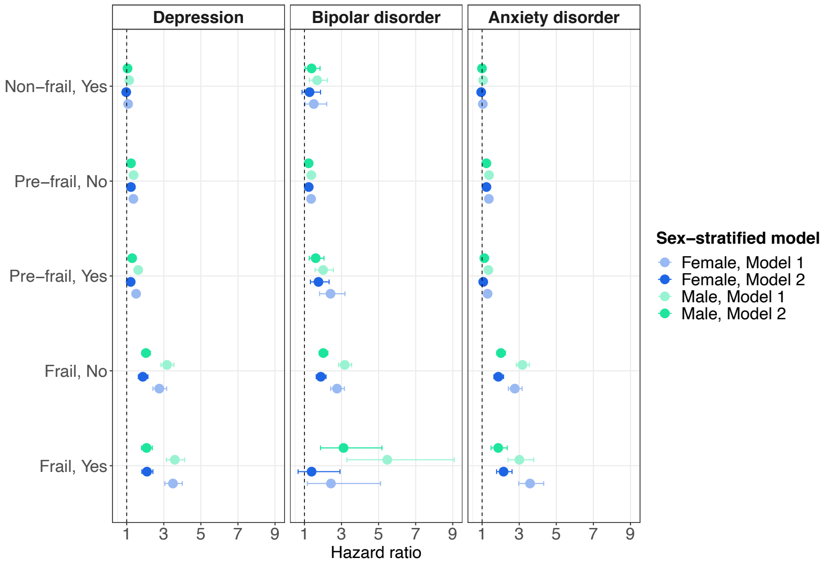

Figure S11. All-cause mortality by frailty phenotype (non-frail, pre-frail and frail) in individuals with and without mental disorders stratified by sex. Reference group: non-frail individuals without mental disorders. Hazard ratios (HR) with 95% confidence intervals from Cox proportional hazards models. Age (in years) was used as the underlying time axis. Model 1 – unadjusted; Model 2 – adjusted for ethnicity, highest qualification, Townsend deprivation index, cohabitation with spouse/partner, smoking status, alcohol intake frequency, systolic and diastolic blood pressure, body mass index, cholesterol, multimorbidity count and assessment centre.

Table S9. All-cause mortality by frailty phenotype in individuals with and without mental disorders stratified by sex

		Males												Females											
		Model 1						Model 2						Model 1						Model 2					
**Depression**		HR	95% CI		$p_{Bonf.}$	p_{BH}	RD %	HR	95% CI		$p_{Bonf.}$	p_{BH}	RD %	HR	95% CI		$p_{Bonf.}$	p_{BH}	RD %	HR	95% CI		$p_{Bonf.}$	p_{BH}	RD %
Non-frail	No	Ref	-	-	-	-		Ref	-	-	-	-		Ref	-	-	-	-		Ref	-	-	-	-	
Non-frail	Yes	1.139	1.057	1.228	0.019	<0.001		1.046	0.970	1.129	>0.999	0.256		1.076	0.991	1.168	>0.999	0.104		0.975	0.897	1.060	>0.999	0.555	
Pre-frail	No	1.372	1.313	1.434	<0.001	<0.001		1.232	1.178	1.288	<0.001	<0.001		1.361	1.285	1.443	<0.001	<0.001		1.230	1.160	1.304	<0.001	<0.001	
Pre-frail	Yes	1.621	1.516	1.734	<0.001	<0.001	66.92	1.295	1.208	1.388	<0.001	<0.001	27.26	1.509	1.402	1.623	<0.001	<0.001	40.81	1.216	1.127	1.312	<0.001	<0.001	-6.01
Frail	No	3.176	2.845	3.546	<0.001	<0.001		2.036	1.817	2.282	<0.001	<0.001		2.759	2.413	3.153	<0.001	<0.001		1.870	1.628	2.148	<0.001	<0.001	
Frail	Yes	3.597	3.139	4.122	<0.001	<0.001	19.33	2.070	1.797	2.384	<0.001	<0.001	3.30	3.496	3.055	4.001	<0.001	<0.001	41.93	2.094	1.817	2.413	<0.001	<0.001	25.71
**Bipolar disorder**		HR	95% CI		$p_{Bonf.}$	p_{BH}		HR	95% CI		$p_{Bonf.}$	p_{BH}		HR	95% CI		$p_{Bonf.}$	p_{BH}		HR	95% CI		$p_{Bonf.}$	p_{BH}	
Non-frail	No	Ref	-	-	-	-		Ref	-	-	-	-		Ref	-	-	-	-		Ref	-	-	-	-	
Non-frail	Yes	1.686	1.268	2.241	0.010	<0.001		1.385	1.041	1.843	0.758	0.028		1.507	1.031	2.203	>0.999	0.044		1.278	0.874	1.870	>0.999	0.247	
Pre-frail	No	1.372	1.313	1.434	<0.001	<0.001		1.230	1.176	1.286	<0.001	<0.001		1.362	1.285	1.443	<0.001	<0.001		1.233	1.162	1.308	<0.001	<0.001	
Pre-frail	Yes	2.009	1.575	2.562	<0.001	<0.001	170.79	1.609	1.260	2.054	0.004	<0.001	165.05	2.408	1.819	3.186	<0.001	<0.001	289.17	1.758	1.325	2.332	0.003	<0.001	225.47
Frail	No	3.175	2.844	3.545	<0.001	<0.001		2.018	1.799	2.264	<0.001	<0.001		2.761	2.415	3.156	<0.001	<0.001		1.883	1.636	2.167	<0.001	<0.001	
Frail	Yes	5.468	3.294	9.079	<0.001	<0.001	105.45	3.110	1.868	5.178	<0.001	<0.001	107.21	2.431	1.158	5.107	0.568	0.026	-18.70	1.386	0.658	2.919	>0.999	0.418	-56.24
**Anxiety disorder**		HR	95% CI		$p_{Bonf.}$	p_{BH}		HR	95% CI		$p_{Bonf.}$	p_{BH}		HR	95% CI		$p_{Bonf.}$	p_{BH}		HR	95% CI		$p_{Bonf.}$	p_{BH}	
Non-frail	No	Ref	-	-	-	-		Ref	-	-	-	-		Ref	-	-	-	-		Ref	-	-	-	-	
Non-frail	Yes	1.053	0.952	1.165	>0.999	0.326		0.991	0.895	1.097	>0.999	0.863		1.038	0.935	1.153	>0.999	0.501		0.949	0.854	1.055	>0.999	0.372	
Pre-frail	No	1.372	1.313	1.434	<0.001	<0.001		1.229	1.175	1.286	<0.001	<0.001		1.362	1.285	1.443	<0.001	<0.001		1.232	1.161	1.307	<0.001	<0.001	
Pre-frail	Yes	1.339	1.214	1.476	<0.001	<0.001	-9.01	1.119	1.014	1.236	0.767	0.028	-47.92	1.293	1.173	1.426	<0.001	<0.001	-18.91	1.060	0.960	1.172	>0.999	0.288	-73.98
Frail	No	3.175	2.844	3.545	<0.001	<0.001		2.012	1.794	2.256	<0.001	<0.001		2.761	2.415	3.156	<0.001	<0.001		1.875	1.631	2.156	<0.001	<0.001	
Frail	Yes	3.008	2.394	3.779	<0.001	<0.001	-7.69	1.866	1.481	2.352	<0.001	<0.001	-14.40	3.584	2.974	4.319	<0.001	<0.001	46.74	2.157	1.780	2.614	<0.001	<0.001	32.18

Note: HR = hazard ratio; CI = confidence interval; RD % = percentage risk difference; Bonf. = Bonferroni; BH = Benjamini & Hochberg. Age (in years) was used as the underlying time axis. Model 1 – unadjusted; Model 2 – adjusted for ethnicity, highest qualification, Townsend deprivation index, cohabitation with spouse/partner, smoking status, alcohol intake frequency, systolic and diastolic blood pressure, body mass index, cholesterol, multimorbidity count and assessment centre.

All-cause mortality by frailty index categories and case status stratified by sex

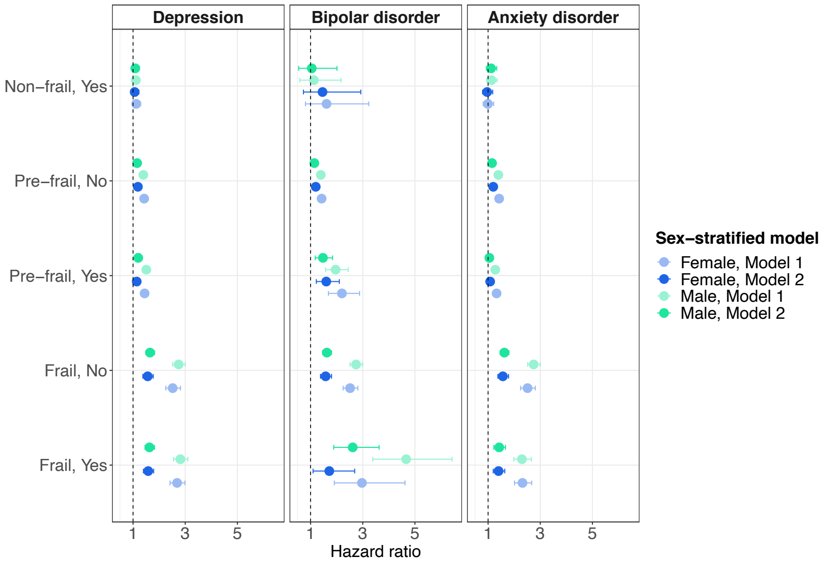

Figure S12. All-cause mortality by frailty index categories (non-frail, pre-frail and frail) in individuals with and without mental disorders stratified by sex. Reference group: non-frail individuals without mental disorders. Hazard ratios (HR) with 95% confidence intervals from Cox proportional hazards models. Age (in years) was used as the underlying time axis. Model 1 – unadjusted; Model 2 – adjusted for ethnicity, highest qualification, Townsend deprivation index, cohabitation with spouse/partner, smoking status, alcohol intake frequency, systolic and diastolic blood pressure, body mass index, cholesterol, multimorbidity count and assessment centre.

Table S10. All-cause mortality by frailty index categories in individuals with and without mental disorders stratified by sex

		Males												Females											
		Model 1						Model 2						Model 1						Model 2					
**Depression**		HR	95% CI		$p_{Bonf.}$	p_{BH}	RD %	HR	95% CI		$p_{Bonf.}$	p_{BH}	RD %	HR	95% CI		$p_{Bonf.}$	p_{BH}	RD %	HR	95% CI		$p_{Bonf.}$	p_{BH}	RD %
Non-frail	No	Ref	-	-	-	-		Ref	-	-	-	-		Ref	-	-	-	-		Ref	-	-	-	-	
Non-frail	Yes	1.107	0.970	1.264	>0.999	0.165		1.085	0.950	1.239	>0.999	0.259		1.120	0.972	1.291	>0.999	0.146		1.062	0.921	1.224	>0.999	0.440	
Pre-frail	No	1.393	1.326	1.464	<0.001	<0.001		1.155	1.093	1.221	<0.001	<0.001		1.425	1.333	1.522	<0.001	<0.001		1.185	1.102	1.275	<0.001	<0.001	
Pre-frail	Yes	1.508	1.410	1.613	<0.001	<0.001	29.29	1.197	1.113	1.288	<0.001	<0.001	27.15	1.443	1.334	1.560	<0.001	<0.001	4.25	1.140	1.047	1.242	0.076	0.003	-24.31
Frail	No	2.747	2.517	2.998	<0.001	<0.001		1.649	1.490	1.825	<0.001	<0.001		2.520	2.253	2.818	<0.001	<0.001		1.561	1.374	1.773	<0.001	<0.001	
Frail	Yes	2.813	2.552	3.101	<0.001	<0.001	3.79	1.629	1.458	1.821	<0.001	<0.001	-3.06	2.688	2.417	2.989	<0.001	<0.001	11.08	1.581	1.396	1.789	<0.001	<0.001	3.47
**Bipolar disorder**		HR	95% CI		$p_{Bonf.}$	p_{BH}		HR	95% CI		$p_{Bonf.}$	p_{BH}		HR	95% CI		$p_{Bonf.}$	p_{BH}		HR	95% CI		$p_{Bonf.}$	p_{BH}	
Non-frail	No	Ref	-	-	-	-		Ref	-	-	-	-		Ref	-	-	-	-		Ref	-	-	-	-	
Non-frail	Yes	1.127	0.586	2.170	>0.999	0.744		1.045	0.543	2.012	>0.999	0.896		1.612	0.804	3.230	>0.999	0.206		1.457	0.727	2.924	>0.999	0.321	
Pre-frail	No	1.392	1.325	1.463	<0.001	<0.001		1.144	1.081	1.211	<0.001	<0.001		1.423	1.331	1.521	<0.001	<0.001		1.198	1.112	1.290	<0.001	<0.001	
Pre-frail	Yes	1.959	1.572	2.442	<0.001	<0.001	144.45	1.474	1.179	1.842	0.020	<0.001	227.81	2.201	1.684	2.878	<0.001	<0.001	184.06	1.601	1.220	2.101	0.021	<0.001	204.18
Frail	No	2.745	2.515	2.995	<0.001	<0.001		1.624	1.463	1.802	<0.001	<0.001		2.515	2.248	2.813	<0.001	<0.001		1.577	1.380	1.803	<0.001	<0.001	
Frail	Yes	4.664	3.384	6.427	<0.001	<0.001	110.00	2.616	1.886	3.628	<0.001	<0.001	159.07	2.969	1.909	4.619	<0.001	<0.001	30.02	1.717	1.096	2.690	0.546	0.024	24.32
**Anxiety disorder**		HR	95% CI		$p_{Bonf.}$	p_{BH}		HR	95% CI		$p_{Bonf.}$	p_{BH}		HR	95% CI		$p_{Bonf.}$	p_{BH}		HR	95% CI		$p_{Bonf.}$	p_{BH}	
Non-frail	No	Ref	-	-	-	-		Ref	-	-	-	-		Ref	-	-	-	-		Ref	-	-	-	-	
Non-frail	Yes	1.129	0.945	1.348	>0.999	0.218		1.114	0.933	1.331	>0.999	0.259		0.992	0.812	1.212	>0.999	0.940		0.962	0.787	1.176	>0.999	0.728	
Pre-frail	No	1.393	1.326	1.464	<0.001	<0.001		1.151	1.088	1.217	<0.001	<0.001		1.422	1.331	1.520	<0.001	<0.001		1.197	1.112	1.289	<0.001	<0.001	
Pre-frail	Yes	1.275	1.165	1.394	<0.001	<0.001	-30.12	1.047	0.953	1.151	>0.999	0.360	-68.56	1.325	1.206	1.456	<0.001	<0.001	-23.02	1.078	0.975	1.192	>0.999	0.173	-60.54
Frail	No	2.747	2.517	2.998	<0.001	<0.001		1.623	1.464	1.799	<0.001	<0.001		2.513	2.247	2.810	<0.001	<0.001		1.566	1.375	1.784	<0.001	<0.001	
Frail	Yes	2.296	1.983	2.658	<0.001	<0.001	-25.81	1.425	1.220	1.666	<0.001	<0.001	-31.72	2.318	2.013	2.670	<0.001	<0.001	-12.86	1.401	1.198	1.639	<0.001	<0.001	-29.18

Note: HR = hazard ratio; CI = confidence interval; RD % = percentage risk difference; Bonf. = Bonferroni; BH = Benjamini & Hochberg. Age (in years) was used as the underlying time axis. Model 1 – unadjusted; Model 2 – adjusted for ethnicity, highest qualification, Townsend deprivation index, cohabitation with spouse/partner, smoking status, alcohol intake frequency, systolic and diastolic blood pressure, body mass index, cholesterol, multimorbidity count and assessment centre.

Sensitivity analysis

Individuals with or without comorbid depression and anxiety disorders

Table S11. Descriptive statistics follow-up, comorbidity depression and anxiety disorders

		**Depression without anxiety disorder**		**Anxiety disorder without depression**		**Comorbid depression and anxiety disorder**	
Full sample size (cases)		283165 (48737)		248495 (14067)		244506 (23712)	
Person-years follow-up		3351539		2950320		2899257	
Median (IQR) censored		12.10 (1.35)		12.19 (1.31)		12.14 (1.32)	
Deaths, all (cases)		16236 (2823)		14261 (848)		14204 (1102)	
**Frailty phenotype**		Censored	Deaths	Censored	Deaths	Censored	Deaths
Non-frail	No	127228	6666	127228	6666	119473	6507
Non-frail	Yes	21989	1055	7064	386	10120	437
Pre-frail	No	90285	6160	90285	6160	84865	6016
Pre-frail	Yes	22063	1472	5854	397	11410	538
Frail	No	3502	587	3502	587	3354	579
Frail	Yes	1862	296	301	65	1080	127
**Frailty index categories**							
Non-frail	No	89247	3513	89247	3513	84753	3436
Non-frail	Yes	10097	349	3162	129	3246	104
Pre-frail	No	125561	8822	125561	8822	117045	8603
Pre-frail	Yes	31569	1870	9133	599	16156	693
Frail	No	6207	1078	6207	1078	5894	1063
Frail	Yes	4248	604	924	120	3208	305

Note: IQR = interquartile range.

Table S12. All-cause mortality by frailty phenotype, comorbidity depression and anxiety disorders

		Model 1						Model 2					
**Depression without anxiety disorder**		HR	95% CI		$p_{Bonf.}$	p_{BH}	RD %	HR	95% CI		$p_{Bonf.}$	p_{BH}	RD %
Non-frail	No	Ref	-	-	-	-	-	Ref	-	-	-	-	-
Non-frail	Yes	1.101	1.032	1.175	0.113	0.005	-	1.069	1.001	1.141	>0.999	0.057	-
Pre-frail	No	1.353	1.307	1.401	<0.001	<0.001	-	1.236	1.193	1.280	<0.001	<0.001	-
Pre-frail	Yes	1.640	1.550	1.735	<0.001	<0.001	81.23	1.413	1.333	1.497	<0.001	<0.001	74.89
Frail	No	2.885	2.651	3.139	<0.001	<0.001	-	1.983	1.817	2.164	<0.001	<0.001	-
Frail	Yes	3.557	3.166	3.996	<0.001	<0.001	35.68	2.212	1.961	2.494	<0.001	<0.001	23.29
**Anxiety disorder without depression**													
Non-frail	No	Ref	-	-	-	-	-	Ref	-	-	-	-	-
Non-frail	Yes	0.992	0.896	1.099	>0.999	0.883	-	1.011	0.912	1.121	>0.999	0.860	-
Pre-frail	No	1.353	1.307	1.401	<0.001	<0.001	-	1.234	1.191	1.278	<0.001	<0.001	-
Pre-frail	Yes	1.263	1.141	1.398	<0.001	<0.001	-25.56	1.175	1.061	1.300	0.059	0.003	-25.43
Frail	No	2.885	2.652	3.139	<0.001	<0.001	-	1.970	1.804	2.151	<0.001	<0.001	-
Frail	Yes	3.444	2.697	4.396	<0.001	<0.001	29.64	2.455	1.920	3.140	<0.001	<0.001	50.10
**Comorbid depression and anxiety disorder**													
Non-frail	No	Ref	-	-	-	-	-	Ref	-	-	-	-	-
Non-frail	Yes	0.984	0.893	1.084	>0.999	0.794	-	0.955	0.866	1.054	>0.999	0.400	-
Pre-frail	No	1.352	1.305	1.400	<0.001	<0.001	-	1.230	1.187	1.275	<0.001	<0.001	-
Pre-frail	Yes	1.199	1.098	1.309	0.002	<0.001	-43.46	1.047	0.958	1.145	>0.999	0.361	-79.46
Frail	No	2.864	2.630	3.118	<0.001	<0.001	-	1.948	1.783	2.129	<0.001	<0.001	-
Frail	Yes	2.892	2.426	3.447	<0.001	<0.001	1.52	1.885	1.577	2.254	<0.001	<0.001	-6.65

Note: HR = hazard ratio; CI = confidence interval; RD % = percentage risk difference; Ref = reference group; Bonf. = Bonferroni; BH = Benjamini & Hochberg. Age (in years) was used as the underlying time axis. Model 1 – unadjusted; Model 2 – adjusted for sex, ethnicity, highest qualification, Townsend deprivation index, cohabitation with spouse/partner, smoking status, alcohol intake frequency, systolic and diastolic blood pressure, body mass index, cholesterol, multimorbidity count and assessment centre.

Table S13. All-cause mortality by frailty index categories, comorbidity depression and anxiety disorders

		Model 1						Model 2					
**Depression without anxiety disorder**		HR	95% CI		$p_{Bonf.}$	p_{BH}	RD %	HR	95% CI		$p_{Bonf.}$	p_{BH}	RD %
Non-frail	No	Ref	-	-	-	-	-	Ref	-	-	-	-	-
Non-frail	Yes	1.082	0.969	1.208	>0.999	0.195	-	1.108	0.992	1.237	>0.999	0.085	-
Pre-frail	No	1.372	1.319	1.427	<0.001	<0.001	-	1.151	1.101	1.202	<0.001	<0.001	-
Pre-frail	Yes	1.521	1.438	1.608	<0.001	<0.001	39.99	1.275	1.200	1.355	<0.001	<0.001	82.72
Frail	No	2.522	2.355	2.700	<0.001	<0.001	-	1.598	1.476	1.730	<0.001	<0.001	-
Frail	Yes	2.849	2.613	3.106	<0.001	<0.001	21.47	1.736	1.576	1.912	<0.001	<0.001	23.03
**Anxiety disorder without depression**													
Non-frail	No	Ref	-	-	-	-	-	Ref	-	-	-	-	-
Non-frail	Yes	0.990	0.831	1.181	>0.999	0.914	-	1.036	0.869	1.235	>0.999	0.744	-
Pre-frail	No	1.371	1.318	1.426	<0.001	<0.001	-	1.151	1.101	1.203	<0.001	<0.001	-
Pre-frail	Yes	1.286	1.179	1.402	<0.001	<0.001	-22.97	1.143	1.045	1.250	0.104	0.005	-5.03
Frail	No	2.519	2.353	2.697	<0.001	<0.001	-	1.577	1.455	1.710	<0.001	<0.001	-
Frail	Yes	2.002	1.669	2.401	<0.001	<0.001	-34.04	1.410	1.170	1.700	0.009	<0.001	-28.96
**Comorbid depression and anxiety disorder**													
Non-frail	No	Ref	-	-	-	-	-	Ref	-	-	-	-	-
Non-frail	Yes	1.040	0.856	1.264	>0.999	0.744	-	1.071	0.881	1.302	>0.999	0.565	-
Pre-frail	No	1.389	1.335	1.446	<0.001	<0.001	-	1.165	1.114	1.219	<0.001	<0.001	-
Pre-frail	Yes	1.146	1.056	1.244	0.031	0.001	-62.41	1.005	0.923	1.095	>0.999	0.914	-97.00
Frail	No	2.561	2.391	2.744	<0.001	<0.001	-	1.594	1.469	1.730	<0.001	<0.001	-
Frail	Yes	2.167	1.928	2.437	<0.001	<0.001	-25.23	1.397	1.232	1.584	<0.001	<0.001	-33.11

Note: HR = hazard ratio; CI = confidence interval; RD % = percentage risk difference; Ref = reference group; Bonf. = Bonferroni; BH = Benjamini & Hochberg. Age (in years) was used as the underlying time axis. Model 1 – unadjusted; Model 2 – adjusted for sex, ethnicity, highest qualification, Townsend deprivation index, cohabitation with spouse/partner, smoking status, alcohol intake frequency, systolic and diastolic blood pressure, body mass index, cholesterol, multimorbidity count and assessment centre.
